# Quality of Life, Social Networking, and Mental Health: Generational Differentiation and Uniqueness in the Context of South Korea

**DOI:** 10.3390/jcm14196739

**Published:** 2025-09-24

**Authors:** Geiguen Shin, Jimin Chae, Yong-Chan Rhee, Youngbin Lym

**Affiliations:** 1 Department of Public Administration, Kookmin University, Seoul 02707, Republic of Korea; gshin@kookmin.ac.kr (G.S.); yblym0207@kookmin.ac.kr (Y.L.); 2 The Convergence Institute of Healthcare and Medical Science, College of Medicine, Catholic Kwandong University, Incheon 22711, Republic of Korea; 3 Department of Geography, Sungshin Women’s University, Seoul 02844, Republic of Korea; 4 Criminology Department, DePaul University, Chicago, IL 60614, USA

**Keywords:** health-related quality of life, social networking, relative deprivation, generational gap in mental health

## Abstract

**Background/Objectives**: Mental health disparities across generations are a growing concern in rapidly changing societies. While health-related quality of life (HRQoL) and social networking are widely recognized as determinants of psychological well-being, less is known about how their effects differ across age groups. This study investigates the generational patterns in the relationship between HRQoL, social networking, and mental health in South Korea. **Methods**: We analyzed data from the nationally representative 2023 Community Health Survey (South Korea). HRQoL, social networking activity, and self-reported mental health outcomes (stress and depression) were assessed. Multivariate models were used to test main effects and interactions, with stratification by generational cohorts. **Results**: Across all models (i.e., all age groups), good HRQoL strongly predicts lower depression (PHQ-9 scores), showing coefficients ranging from –1.20 to –1.48, *p* < 0.001. Social networking activity also predicts reduced depressive symptoms, with significant effects from the thirties onward (e.g., –0.317 in the 30s, –0.507 in the 50s, –0.424 in the 70s +; all *p* < 0.001). However, the interaction term between HRQoL and social networking activity yields unexpected findings. The interaction becomes positive and significant, with coefficients that increase steadily by age: 0.388 in the 40s, 0.472 in the 50s, 0.533 in the 60s, and 0.638 in the 70s + (all *p* < 0.001). Using stress (1 = high-level, 0 = low-level) as the outcome variable, with the same set of covariates, it replicates the findings similar to those obtained when PHQ-9 as the outcome variable. **Conclusions**: The results suggest that the protective role of HRQoL and social networking is not uniform across generations. In South Korea, relative deprivation and social comparison may intensify with age, amplifying the psychological burden despite higher quality of life or social participation. These findings highlight the need for mental health interventions and policy responses that account for generational differences in the social determinants of well-being.

## 1. Introduction

Mental disorders remain among the leading causes of global disease burden, with the Global Burden of Disease (GBD) 2019 analysis showing no reduction in rank since 1990 and estimating more than 125 million disability-adjusted life years (DALYs) attributable to mental disorders [1,2]. Complementary re-analyses indicate that, by 2025, mental health conditions account for 290 million DALYs—183 million directly and 107 million indirectly by worsening other noncommunicable diseases [3,4]. Evidence from the COVID-19 pandemic indicates additional deterioration—WHO estimated a 25% global increase in anxiety and depression in 2020, and recent lifespan analyses show especially high incidence and years lived with disability among adolescents and young adults [5,6,7]. These trends highlight the urgent need to identify upstream, modifiable determinants of psychological well-being.

Against this backdrop, health-related quality of life (HRQoL)—a multidimensional construct capturing perceived physical, mental, emotional, and social functioning—has become a core outcome and policy target because it reflects how health states are experienced in daily life and predicts morbidity, health service utilization, and mortality [8,9,10]. Widely used generic instruments such as the EQ-5D (covering mobility, self-care, usual activities, pain/discomfort, anxiety/depression) and the SF-36/SF-12 (yielding physical and mental component summaries) operationalize HRQoL with strong evidence supporting their validity and responsiveness across diverse populations [11,12,13,14].

A substantial body of literature links lower HRQoL with poorer mental health—particularly depressive and anxiety symptoms—across community and clinical samples. Systematic reviews examine both cross-sectional and longitudinal associations, with several studies reporting bidirectional effects whereby deteriorations in HRQoL forecast subsequent depression/anxiety and vice versa [15,16]. For example, in longitudinal cohorts of adults, lower HRQoL prospectively predicts higher depression and anxiety, while depressive symptoms in turn erode HRQoL, consistent with mutually reinforcing dynamics [16,17]. Recent work across diverse conditions (e.g., post-COVID syndrome, migraine, student populations) and contexts consistently finds that diminished HRQoL accompanies greater psychological morbidity, whereas improvements in functioning track better mental health outcomes [18,19,20]. Moreover, interventions that enhance HRQoL—such as exercise and other non-pharmacological strategies among older adults—also reduce depressive symptoms, underscoring HRQoL as a practical leverage point [21,22,23]. These bidirectional links motivate analyses that treat HRQoL not merely as a correlate but as a leverage point for prevention and care.

At the same time, important controversies remain. First, the causal direction between HRQoL and mental health is debated: while some evidence suggests HRQoL predicts future mental health, others find the reverse, and several report bidirectional associations—implying potential feedback loops that complicate intervention timing and mediation tests [15]. Second, population heterogeneity is likely. Comorbidity, multimorbidity, and socioeconomic stressors may moderate the HRQoL–mental health link, suggesting the need to examine interactions with social determinants [1,24]. Most longitudinal studies that seek to disentangle the causal direction between HRQoL and mental health remain embedded in a medicalized paradigm: they adjust meticulously for clinical comorbidity and a limited set of demographic covariates but pay less attention to non-medical social determinants such as education, income, neighborhood context, or the structure and quality of people’s social ties. The latter in particular has received limited attention, leaving important pathways linking HRQoL, social networking, and mental health only partially understood.

Social connection—encompassing the structure (e.g., network size, living arrangement), function (received and perceived support, loneliness) and quality (interpersonal ties, social inclusion) of relationships—have moved to the center of global health policy [25]. In June 2025, the WHO Commission on Social Connection reported that about 1 in 6 people experience loneliness and linked loneliness to more than 871,000 deaths annually, and in May 2025, the World Health Assembly adopted its first resolution recognizing social networking and connection as a global health priority [26,27,28]. Meta-analyses show that stronger social relationships confer a 50% increased survival odds, whereas isolation and loneliness are associated with substantially elevated mortality risk, highlighting social networking as a potent, modifiable influence on both mental and physical health [29,30].

A central theoretical question—highly relevant to policy design—is whether social networking buffers the adverse impact of poor HRQoL on mental health and whether this buffering effect varies by age. The classic stress-buffering hypothesis posits that social support attenuates the psychological consequences of stressors and health limitations [31,32]. Evidence in older adults often supports this perspective: higher social support or participation weakens the link between poor perceived health and depressive symptoms, with some studies finding the strongest effects among the “oldest-old” [33,34,35].

Critically, age may shape how social networking interacts with HRQoL to affect mental health, but findings diverge. Research in lifespan development and gerontology indicates that social resources gain importance with age—consistent with socioemotional selectivity theory and the cumulative impact of health stressors—suggesting that the buffering effect of social networking on the HRQoL–mental health pathway may be stronger in later life. Major reports suggest that social isolation and loneliness are widespread, harmful, and insufficiently addressed among adults aged 50 and older [36]. Because older adults face cumulative health stressors and losses, high-quality close ties may be especially protective against depressive symptoms and functional decline—up to the point where sustained stressors overwhelm resources [37]. Policy bodies emphasize targeting connection in older age due to its substantial population burden and consequences [36,37].

In contrast, longitudinal and ecological studies document that loneliness is not confined to older adults: it often peaks in young adulthood, declines through midlife, and rises again in the oldest-old, indicating distinct age-graded risks and mechanisms [38]. Moreover, very large population data during 2020–2021 show the association between loneliness and depressive symptoms was stronger in younger than in older adults, suggesting that, for mental health outcomes, social networking and connection deficits may exert especially larger effects on depressive/anxiety symptoms. Thus, high social networking should more strongly reduce symptom risk in younger groups [39,40]. Together, these patterns motivate explicit tests of age-differentiated moderation, rather than assuming uniform effects across the life course.

This study examines how HRQoL affects mental health and whether social networking moderates this association differentially by age. Advancing prior work, we integrate the validated HRQoL measure with social networking and test competing age-pattern hypotheses grounded in lifespan theory and recent population evidence. Specifically, we posit:

**Hypothesis** **1** **(Main effects).** *Lower HRQoL (and/or weaker social networking) will be associated with poorer mental health (higher stress/depressive symptoms).*

**Hypothesis** **2** **(Buffering: moderation regardless of age).** *Higher social networking will attenuate the negative association between low HRQoL and mental health across all ages; we refer to this as the buffering effect in this study.*

**Hypothesis** **3a** **(Younger-stronger moderation).** *The buffering effect of social networking on the HRQoL–mental health link will be stronger among younger adults, reflecting their heightened sensitivity of depressive symptoms to loneliness.*

**Hypothesis** **3b** **(Older-stronger moderation).** *Alternatively, consistent with socioemotional selectivity and cumulative stress exposure, the buffering effect will be stronger among older adults.*

By adjudicating these diverging hypotheses, the study aims to clarify when and for whom social networking most effectively offsets the mental health consequences of diminished HRQoL. The modeling strategies are intended to examine age-targeted prevention and intervention strategies aligning with the WHO Commission’s call to embed social cohesion within mental health policy. In brief, our hypotheses present that HRQoL may be strongly related to mental health and that social networking activities may buffer this association; the magnitude of buffering may vary by age, with policy implications for prioritizing connection-building strategies at life stages where they may yield the largest mental-health returns.

The purpose of this study is to examine how HRQoL affects mental health and whether social networking moderates this relationship across age groups. We expect that lower HRQoL will predict poorer mental health, while stronger social networking will buffer against these effects. Given mixed evidence, we test competing hypotheses: that the protective role of social networking is stronger in younger adults versus in older adults. By adjudicating between these views, the study clarifies when and for whom social networking most effectively supports mental health.

## 2. Materials and Methods

This study uses the Community Health Survey (CHS) 2023 in South Korea, an official nationwide health survey conducted annually by the Korea Disease Control and Prevention Agency (KDCA) (KDCA is a governmental agency in South Korea similar to Centers for Disease Control and Prevention (CDC) in the United States (U.S.)). The program used is Stata. The collection of CHS is mandated by the Regional Public Health Act of South Korea. This survey dataset has been collected at each public health center in South Korea to provide essential evidence for policymaking [41]. We used the CHS 2023 dataset instead of the CHS 2024 dataset, since it is the most recent release that contains both HRQoL and social networking measures.

The first outcome variable used in this study is “depression,” measured using the Patient Health Questionnaire (PHQ-9), consistent with past studies [42,43]. In the CHS 2023 dataset, nine survey items of depression are coded from 1 (“not at all”) to 4 (“almost every day”). We summed up all nine questions asking about depression signs and subtracted nine to compose PHQ-9, which we refer to as “depression” in this study. This is because participants who replied “not at all” scored a total of “9×1 = 9” in the CHS 2023. As shown in Table 1, the lowest value of the depression (PHQ-9) is “0,” and the highest score of the depression (PHQ-9) equals “27,” successfully creating the dependent variable of interest.

The second outcome variable is “stressed.” It equals “1” if participants reported experiencing a high-level of mental stress and “0” if they reported a low-level of mental stress. This measure is based on a 4-point Likert scale item in the CHS 2023 dataset, where 1 = “almost always,” 2 = “often,” 3 = “not so much,” and 4 = “rarely.” Using this item, we constructed a binary variable of stress: “1” if participants answered, “almost always” or “often,” and “0” if they answered, “not so much” or “rarely.”

To measure social networking, it is important to address structural indicators, such as participation in social networking activities and capture the tangible opportunities individuals have to interact [26]. The CHS 2023 dataset asks survey participants whether they engage in any in-person social networking activity at least once per month (excluding online activities). If the participants replied “yes” to this question, we have coded them as “1.” If they replied “no” to this question, we have coded them as “0.” This single-item measure serves as a valid proxy for the structural component of social networking, as it directly reflects active involvement in a social context.

Relatedly, the CHS 2023 dataset treats HRQoL as the subjective feeling and perception of survey participants’ health [41], rather than more objective health survey measures, such as stroke and diabetes [9]. It asks participants to rate their subjective health condition in daily life, from “very bad” to “very good.” In this study, the primary variable of interest is the interaction between “social networking activity” and “HRQoL,” used to examine whether social networking moderates the relationship between HRQoL and mental health (PHQ-9).

In addition to the two primary independent variables, we have included a wide range of variables available in the survey dataset. The list of all variables, including controls, is available in Table 1 (Summary of Statistics). For example, we included survey items, including but not limited to whether participants have received health check-ups; had diabetes or a stroke; recognized early signs of a heart attack; had access to medical services; their living conditions and environment, socioeconomic status, high blood pressure, height, weight, exercise habits; contact with family, relatives, and/or friends; smoking behavior; experiences of accidents or addiction; housing type; neighborhood safety; educational attainment; and marital status.

We employ linear fixed-effects models with district-level fixed effects clustered by district, to account for unobserved heterogeneity such as variation in the quality of public health centers. All districts are completely nested within provinces in South Korea. For robustness checks, we use inverse probability weighting (IPW) models, where we use logistic models to specify $\hat{P}(T_{i}=1|X_{i})$. In the IPW framework, the weight of the treated group is given as ${\hat{w}}_{1}=\frac{1}{\hat{P}(T_{i}=1|X_{i})}$. In contrast, the weight of the control group is given as ${\hat{w}}_{0}=\frac{1}{1-\hat{P}(T_{i}=1|X_{i})}$. For example, if the $\hat{P}(T_{i}=1|X_{i})$ reaches close to “1,” it means giving less weight to the treated group, since the probability of being assigned to the treated group is already high.

We estimate average treatment effects (ATE) in IPW robustness checks, where $\hat{ATE}=E\left[Y_{i}|T_{i}=1\right]-E\left[Y_{i}|T_{i}=0\right]=\frac{1}{n}\left(\sum_{i=1}^{n}\frac{T_{i}}{\hat{P}(T_{i}=1|X_{i})}Y_{i}\right)-\frac{1}{n}\left(\sum_{i=1}^{n}\frac{1-T_{i}}{1-\hat{P}(T_{i}=1|X_{i})}Y_{i}\right)=E\left[\frac{T_{i}}{\hat{P}(T_{i}=1|X_{i})}Y_{i}\right]-E\left[\frac{{1-T}_{i}}{1-\hat{P}(T_{i}=1|X_{i})}Y_{i}\right]$. A key advantage of IPW is that it adjusts for unobserved correlations with covariates and better accounts for potential data imbalances between treated and control groups, making it suitable for robustness checks [44]. We consider findings robust only when results from both fixed-effects and IPW models converge. This dual approach strengthens causal inference and provides a more reliable understanding of factors that potentially contribute to depression in Korean society. The country has long been known to experience a high level of unhappiness, and this study seeks to provide insights into the patterns of mental depression in each generation.

## 3. Results

Table 2 and Table 3 summarize the main findings from the fixed-effects models. Overall, the results suggest that Hypothesis 1 is supported, but not Hypotheses 2, 3a, and 3b. In fact, contrary to Hypothesis 3b, we find significant evidence in the opposite direction, suggesting a mechanism of relative deprivation rather than buffering.

To be specific, the results in Table 2 indicate that both HRQoL and social networking activities are negatively associated with depressive symptoms, consistent with Hypothesis 1. Across all models (i.e., all age groups), good HRQoL strongly predicts lower PHQ-9 scores (coefficients ranging from −1.20 to −1.48, *p* < 0.001). Social networking activity also predicts reduced depressive symptoms, with significant effects from the thirties onward (e.g., −0.317 in the 30s, −0.507 in the 50s, −0.424 in the 70s +; all *p* < 0.001).

However, the interaction term between HRQoL and social networking activity yields unexpected findings. The interaction term is positively and significantly associated with a higher likelihood of experiencing depression (PHQ-9) among older generations (Columns 3–6), intensifying relative deprivation. Such effects remain insignificant among younger generations (Generations in South Korea are counted in 10-year intervals (e.g., 20s, 30s, etc.) [45,46]).

Specifically, we present the results of this study here. Among individuals in their twenties (Column 1) and thirties (Column 2), the interaction is statistically insignificant, providing no evidence for Hypothesis 3a (younger-stronger moderation). Simultaneously, results suggest that younger populations are less susceptible to the social comparative mindset prevalent in Korean society.

By contrast, among older adults, the interaction becomes positive and significant, with coefficients that increase steadily by age: 0.388 in the 40s, 0.472 in the 50s, 0.533 in the 60s, and 0.638 in the 70s + (all *p* < 0.001). These findings contradict Hypothesis 3b, which anticipated stronger buffering effects in later life. Instead, they suggest that when older adults with higher HRQoL engage in social networking activities, they may experience intensified depressive symptoms, likely due to comparing their lives with others (i.e., social comparison) and relative deprivation.

Model fit statistics are consistent across age groups, with R^2^ values ranging from 0.177 to 0.208, and all models include district fixed effects and a comprehensive set of control variables. Taken together, these results highlight that while HRQoL and social networking, each independently reduces depression risk, their interaction does not produce buffering effects. Rather, among older adults, social networking in the context of good HRQoL appears to exacerbate depressive symptoms.

**Figure 1:**
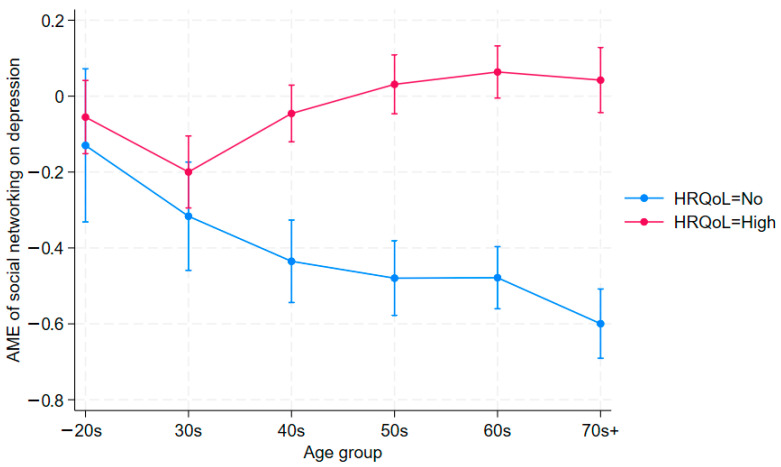
Average Marginal Effects of Social Networking Moderated by HRQoL on Depression (95% C.I.).

**Figure 2:**
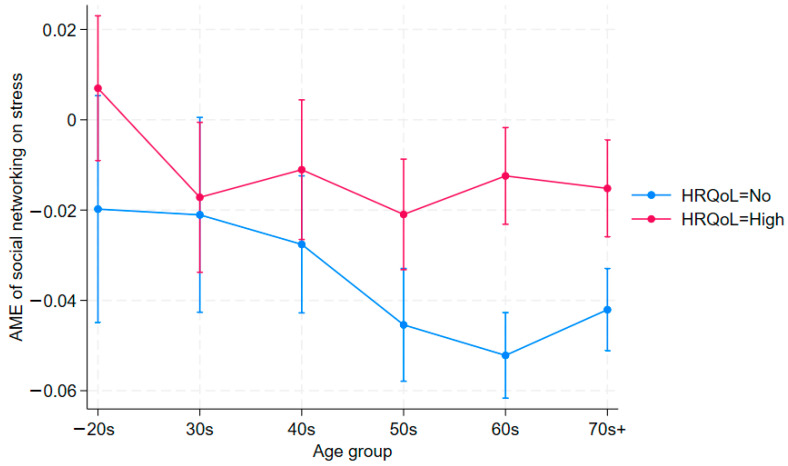
Average Marginal Effects of Social Networking Moderated by HRQoL on Stress (95% C.I.).

To gain deeper insight into the moderating effect of social networking, we plot the average marginal effects of social networking activity by HRQoL across age groups (Figure 1 and Figure 2). In both figures, the blue line (HRQoL = No) serves as the benchmark. A higher average marginal effect (AME) of social networking when HRQoL is high—the red line—means a positive, statistically significant moderating effect on the outcome (depression or stress). The figures corroborate the regression results reported in Table 2 and Table 3. For detailed figures, please see Figures S1 and S2 in the Supplementary Materials. In Figure 1, for example, blue and red lines overlap for those who are in their 20s or younger and those in their 30s, indicating no significant moderation among younger generations.

By contrast, those who are in their 40s and higher, for example, show a widening gap between those who show low quality of life and those who show high quality of life, revealing different patterns compared to younger generations. In these older age groups, individuals with higher HRQoL who engage in social networking are more likely to report depression compared to those who do not. This reversal is consistent with mechanisms of relative deprivation and upward social comparison. Moreover, the magnitude of the interaction effect is larger in older cohorts, suggesting the intensification of these dynamics in later life. This age-differentiated pattern highlights the importance of examining moderation explicitly rather than assuming uniform effects across the life course.

Table 3 presents using stress (1 = high-level, 0 = low-level) as the outcome variable, with the same set of covariates in Table 2. As shown, it replicates the findings in Table 2 using depression (PHQ-9) as the outcome variable. The main effects are consistent with expectations: both good HRQoL and participation in social networking are negatively associated with stress across most age groups. However, as with the depression models (given in Table 2), the interaction terms reveal a different pattern by age. Among younger adults (40s or below), the HRQoL and social networking interaction is statistically insignificant. Conversely, in older cohorts (50s and above), the interaction terms are positive and significant (e.g., 0.023 in the 50s, 0.040 in the 60s, 0.029 in the 70s; all *p* < 0.01), indicating that social networking in combination with higher HRQoL is associated with a greater likelihood of reporting high stress. This reversal parallels the findings in Table 2 and further supports the interpretation of relative deprivation and upward social comparison dynamics in later life [47]. These results remain similar in Figure 2, which is based on Table 3. Figure 2’s results are similar to those in Figure 1, though less pronounced. Since Figure 2 is derived from Table 3, Table 3 provides the more precise estimates; Figure 2 is included for reference. In general, Figure 1 and Figure 2 mirror the patterns in Table 2 and Table 3, respectively.

### Robustness Checks

**Figure 3:**
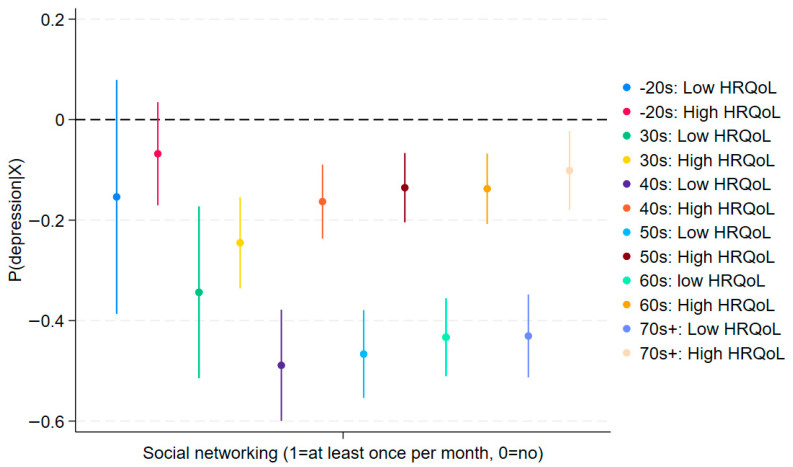
Robustness Check: Moderating Effects on Depression by Age Group using IPW (95% C.I.). ***Note***: A higher expected value of the outcome here means a higher likelihood of experiencing depression (PHQ-9) (see also Table 2). It replicates findings in Table 2.

**Figure 4:**
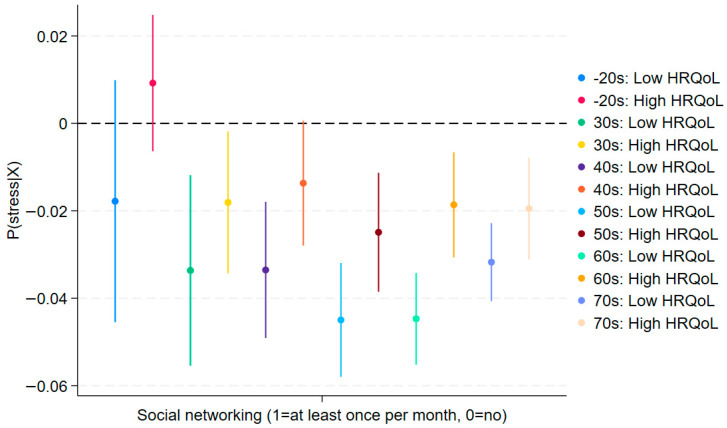
Robustness Check: Moderating Effects on Stress by Age Group using IPW (95% C.I.). ***Note***: It replicates findings in Table 3 and is consistent with those in the depression model (PHQ-9).

**Figure 5:**
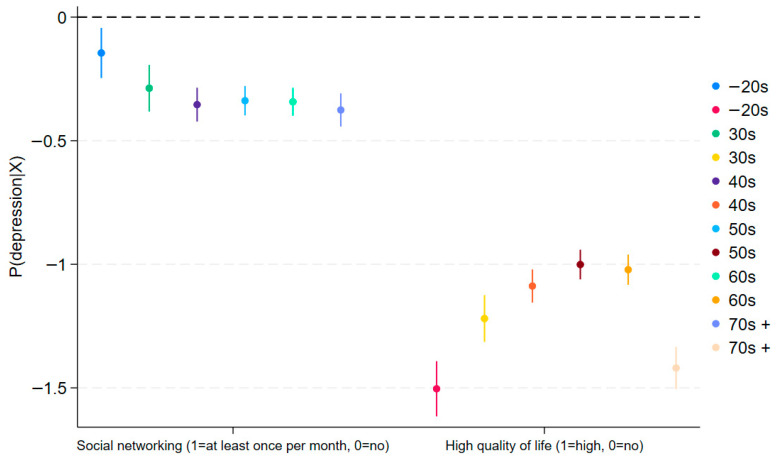
Robustness Check: Main Effects on Depression by Age Group using IPW (95% C.I.).

**Figure 6:**
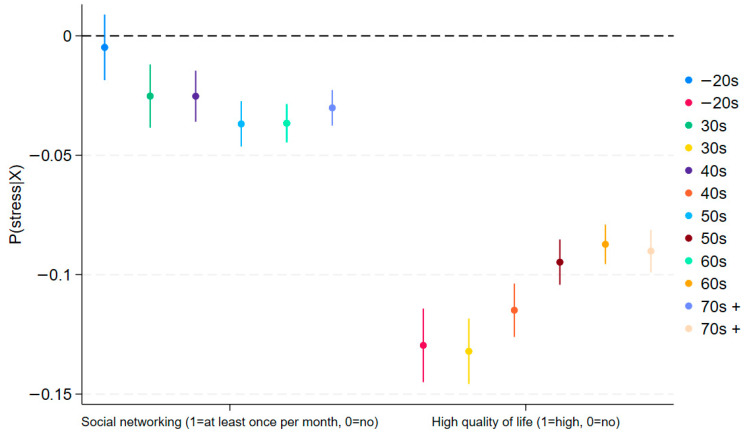
Robustness Check: Main Effects on Stress by Age Group using IPW (95% C.I.).

In Figure 3, Figure 4, Figure 5 and Figure 6, we estimate IPW models with the same set of covariates as in Table 2 and Table 3 to examine whether unobserved correlations in the fixed-effects models alter the key findings. If the IPW models were to return insignificant results for the interaction terms, this would raise concerns about the validity of the fixed-effects estimates. However, in Figure 3, Figure 4, Figure 5 and Figure 6, we confirm that the after-match standardized differences are below the conservative threshold of |±0.1|, indicating good covariate balance (we used provincial-level fixed effects for IPW models only. This is because provincial-level fixed effects are highly correlated with district-level fixed effects, given that a district is completely nested within each province. By design, matching models automatically account for such correlations. We note here one more time that post-match standardized differences are below |±0.1|).

As shown in Figure 3, the slopes for low and high HRQoL largely overlap for participants in their 20s and younger, as well as those in their 30s. The confidence intervals also cross (blue and red lines for the 20s and below, and green and yellow lines for the 30s), suggesting no significant moderation by social networking among younger generations. This corroborates the fixed-effects results that Hypothesis 3a (younger-stronger moderation) is not supported. By contrast, we observe significant moderating effects among older generations. The non-overlapping confidence intervals among 40s and older confirm that this difference is statistically significant, replicating the positive interaction effects observed in Table 2 (Columns 3–6). These results of moderating effects remain similar in Figure 4 (stress). Together, these robustness checks reinforce the conclusion that in later life, social networking coupled with good HRQoL may exacerbate depressive symptoms, consistent with relative deprivation and social comparison mechanisms.

Likewise, the main effects of quality of life and social networking in Figure 5 (depression) and Figure 6 (stress) show outcomes similar to those in Table 2 and Table 3. These findings generally support Hypothesis 1 (main effects). Therefore, we conclude that we find no warning signals from the IPW models, which account more effectively for potential unobserved correlations with the covariates.

## 4. Discussion

The results of this study can be summarized as follows. First, we find moderating effects of HRQoL and social networking activities on both mental stress and depression among older generations, in contrast to younger generations South Korea. In fact, we find unexpected positive coefficients of the moderating effects in this research. Second, we find the fixed-effects models, the main findings of this study, to be reasonably robust based on the IPW modeling results. IPW models were able to replicate the main results from the fixed-effects models, implying that the findings are not driven by unobserved correlations with the covariates.

Relatively few empirical studies have systematically examined generational differences between younger and older populations in terms of who is more likely to suffer from social isolation, despite a rising level of controversy and robust discussions on related topics. Given the significant generational conflicts in South Korea, each generation claims to be suffering more than the others due to their respective and different socioeconomic hardships they have experienced [48,49]. Although this study does not argue that one generation suffers more than another, the results suggest that the combined effects of social networking activities and HRQoL have unexpectedly stronger-and adverse-among older generations.

Not only do these findings contradict the buffering effect hypotheses, but they also challenge common sense and consensus in Korean society. First, the common stereotype against younger generations posits that they are less likely to engage in in-person social networking than ever, compared to the older generations. Perhaps, this may be true due to the speeding technological advancements of social media [50,51]. A study argues that social support is crucial for ensuring a high quality of life, and that close friendships and social networks should contribute to better quality of life, especially among younger generations (46). In this sense, they contend that the key to addressing social issues affecting younger generations—such as high unemployment, some of the lowest fertility rate, and resulting social stress in South Korea—lies in strengthening social support.

Based on this view, we initially hypothesized that the effect of social networking activities, when combined with a better HRQoL, would result in lowering mental stress and depression simultaneously due to buffering effects. For example, social isolation among younger populations [52], also referred to as “Hikikomori,” has become a severe problem in South Korea [48,53]. In fact, mental depression and suicides among youths have been a consistent problem in Korean society [54], and this is why we have assumed that (in-person) social networking activities should have a larger effect on younger generations in Hypothesis 3a [55].

Indeed, the findings of main effects among younger generations show that HRQoL and social networking are significantly associated with both mental stress and depression, similar to a past study [53]. These findings seem to support the claim of the “Hikikmori” phenomenon and stereotype against younger generations. Likewise, the main effects of HRQoL and social networking are likely to reduce the level of depression among older generations, consistent with Hypothesis 1. This also seems to support the claim of social isolation and lonely death issues of older generations.

However, the results of this research show simultaneously that as citizens age in Korean society over time, they become increasingly exposed to the entrenched relative deprivations and comparative mentality in South Korea [48]. In other words, as citizens age, they begin to notice the gaps in health-related quality of life among those who were once considered to belong to the same socioeconomic class or living condition when they were younger. These results suggest how social comparison mentalities prevalent in Korean society are manifested, but especially among older generations [47]. They contradict Hypothesis 3b of buffering effects among older generations.

A part of such a phenomenon could stem from social stigma issues in South Korea [56]. A past study shows that Koreans are more likely to suffer from social stigma if their friends or relatives figure out that a psychiatrist is treating the person, stigmatizing the patient as a mentally problematic person. Even though this perspective helps explain why Koreans are likely to express depression, it does not fully explain the generational gaps found in this research. Alternatively, it could be the case that older generations—despite reporting stronger social ties on the surface (e.g., through family or friends)—experience these interactions as more superficial than emotionally fulfilling. As a result, their social networks may fail to provide the psychological support needed to buffer against mental health stressors, thereby amplifying the negative effects of relative deprivation and social comparison mentalities [47]. As they report higher quality of life, they become more susceptible to relative—rather than absolute—deprivation, since they can compare their living conditions and socioeconomic status with others. In this context, engaging in social networking is associated with greater mental stress and depression.

Even though future studies are needed to provide further alternative explanations or verification of each perspective, we argue that this research offers preliminary guidance for understanding generational differences in social comparison mentality in a country that has experienced substantial economic growth. Since South Korea underwent exceptionally rapid economic development, each generation’s life experiences differ significantly, making it difficult for generations to fully understand one another. We cautiously suggest that the mental health differences identified in this study may also be applicable in other contexts that have experienced rapid social change and/or economic growth.

However, this study is not without limitations. First, this study did not investigate the effects of online networking activities, as its primary objective was to assess the impact of in-person social interactions. The dataset lacked variables capturing online networking behaviors (e.g., participation in virtual communities or online support groups). Given the growing role of digital platforms in shaping social networking and mental health, future research should incorporate both online and offline dimensions to provide a more comprehensive understanding of how different modes of networking influence well-being.

Second, we were unable to examine the multi-year effects of the variables due to inconsistencies in publicly available datasets. We emphasize the need for government agencies to release longitudinal datasets with identical survey items to facilitate temporal comparisons. Even when survey items appear similar, changes in question wording or response categories can prevent researchers from merging datasets across years. For instance, we could not incorporate the CHS 2024 dataset, because it omitted measures of social networking activity behaviors. Ensuring consistency in survey design over time would substantially enhance the capacity of researchers to track trends, assess causal relationships, and produce evidence-based policy recommendations.

Third, while the possibility of reverse causality warrants consideration, we contend that it is unlikely to account for the key finding of this study—particularly the interaction between HRQoL and social networking. From a theoretical standpoint, it is implausible that an increase in depressive symptoms would simultaneously lead to a higher level of HRQoL and stronger social networking. Such a pattern would contradict established evidence linking depression to declines in both quality of life and social engagement. Empirically, our results (see Table 2 and Table 3) show no indication that the observed interaction effect could be driven by such a reverse pathway.

Also, the $R^{2}$ values are relatively small. Even though $R^{2}$ does not serve as an absolute measure of judging whether the model is strong enough or not, it does suggest that there are other factors explaining the variations in mental health other than social comparison mentalities. This is one reason behind applying IPW models. We leave examining other potential factors influencing the mental health outcomes to future research.

Lastly, we argue that future studies should examine additional dimensions of quality of life and social networking—such as the perceived quality, stability, and reciprocity of social ties—rather than focusing solely on their quantity or frequency. Investigating these qualitative aspects may provide deeper insight into how different types of social relationships interact with various components of well-being, potentially revealing mechanisms that remain obscured when only structural measures are considered.

## 5. Conclusions

In sum, our findings challenge the common expectations and hypotheses that better HRQoL and stronger social networking exert buffering effects. Instead, HRQoL and social networking activities appear to have independent effects by decreasing the likelihood of stress and depression. For example, social networking activities decreased mental stress and depression *when* they reported a lower level of HRQoL (i.e., quality of life = 0). Likewise, a higher level of quality of life decreased mental stress and depression *when* they reported they do not engage in social networking activities (i.e., social networking activities = 0) [48,49,53]. By contrast, older generations were more likely to experience mental stress and depression when they reported higher HRQoL and were engaged in in-person social networking activities, likely reflecting dynamics of relative deprivation and upward social comparison that are deeply rooted in Korean society.

## Figures and Tables

**Table 1:** Summary of Statistics.

	(1)	(2)	(3)	(4)	(5)
Variables	N	Mean	S.D.	Min.	Max.
***Dependent Variables***					
Depressed (PHQ-9)	203,320	2.298	3.194	0	27
Stressed (1 = high, 0 = low)	203,320	0.224	0.417	0	1
***Key Independent Variables of Interest***					
Health-related quality of life (1 = high, 0 = no)	203,320	0.385	0.487	0	1
Participate in social networking activities at least once per month (1 = at least once per month, 0 = no)	203,320	0.496	0.500	0	1
***Covariates***					
Age	203,320	55.99	17.449	19	106
Sex (1 = man, 0 = woman)	203,320	0.456	0.498	0	1
Neighborhood type code (1 = Dong, 2 = Eup/Myeon)	203,320	1.425	0.494	1	2
Housing type code (1 = Non-APT, 2 = APT)	203,320	1.459	0.498	1	2
Diverse family or not (1 = yes, 0 = no)	203,320	0.0139	0.117	0	1
Use a safety belt? (1 = don’t drive, 2 = not at all, 3 = rarely, 4 = sometimes, 5 = often, 6 = always)	203,320	3.908	2.399	1	6
High-intensity physical activity in the last week (days)	203,320	0.801	1.677	0	7
Moderate-intensity physical activity in the last week (days)	203,320	1.382	2.166	0	7
Walked more than 10 min a week (days)	203,320	4.263	2.613	0	7
How often did you have your breakfast for the past 1 year (in a week)? (1 = 5–7 days, 2 = 3–4 days, 3 = 1–2 days, 4 = 0 day)	203,320	1.809	1.226	1	4
Height (cm)	202,689	163.9	9.017	50	200
Weight (kg)	203,245	63.93	12.52	20	163
Received influenza vaccine (1 = yes, 0 = no)	203,320	0.597	0.490	0	1
Had high blood pressure (1 = yes, 0 = no)?	203,320	0.311	0.463	0	1
Had diabetes (1 = yes, 0 = no)?	203,320	0.136	0.343	0	1
Havent’ received medical service when needed in the past year (1 = yes, 2 = no, 3 = didn’t need it)?	203,320	2.027	0.360	1	3
Had accident or addicted in the last year (1 = yes, 0 = no)?	203,320	0.0605	0.238	0	1
Trust your neighbor (1 = yes, 0 = no)?	203,320	0.709	0.454	0	1
Neighbors help each other for events (1 = yes, 0 = no)?	203,320	0.485	0.500	0	1
Safe neighborhood (1 = yes, 0 = no)?	203,320	0.867	0.340	0	1
Good natural environment of the neighborhood (1 = yes, 0 = no)?	203,320	0.834	0.372	0	1
Neighborhood equipped with good living conditions (1 = yes, 0 = no)?	203,320	0.857	0.350	0	1
Good access to public transportation (1 = yes, 0 = no)?	203,320	0.706	0.456	0	1
Satisfactory medical service conditions in the neighborhood (1 = yes, 0 = no)?	203,320	0.738	0.440	0	1
How often do you contact your closest family or relative (1 = frequently, 0 = not so much)?	203,320	0.591	0.492	0	1
How often do you contact your closest neighbor (1 = frequently, 0 = not so much)?	203,320	0.505	0.500	0	1
How often do you contact your closest friend (1 = frequently, 0 = not so much)?	203,320	0.520	0.500	0	1
Occupation status (1 = employer, 2 = salaried, 3 = working with no salary, 8 = unemployed)	203,310	4.019	3.021	1	8
Basic livelihood security beneficiary or not (1 = yes, 0 = no)	203,320	0.049	0.215	0	1
Ever smoked (1 = yes, 0 = no)?	203,320	0.387	0.487	0	1
Indirectly smoked (1 = yes, 0 = no)?	203,320	0.003	0.053	0	1
Ever drank alcohol (1 = yes, 0 = no)?	203,318	0.834	0.373	0	1
Was on a diet (1 = yes, 0 = no)?	203,319	0.642	0.479	0	1
Good teeth condition (1 = yes, 0 = no)?	203,320	0.259	0.438	0	1
Ever gambled (1 = yes, 0 = no)?	203,320	0.280	0.449	0	1
Had a health check-up in the past 2 years? (Cronbach’s alpha = 0.77)	203,320	0.697	0.409	0	1
Had a stroke (1 = yes, 0 = no) (Cronbach’s alpha = 0.84)?	203,320	0.812	0.302	0	1
Had early signs of heart attack (1 = yes, 0 = no) (Cronbach’s alpha = 0.79)?	203,320	0.738	0.337	0	1
Had higher education (1 = yes, 0 = no)?	203,284	0.403	0.490	0	1
Married (1 = yes, 0 = no)	203,306	0.666	0.472	0	1

**Note:** All of the covariates in Table 1 were consistently used in all of the models in this study.

**Table 2:** Results of Depression (PHQ-9).

	(1) Depressed (PHQ-9)	(2) Depressed (PHQ-9)	(3) Depressed (PHQ-9)	(4) Depressed (PHQ-9)	(5) Depressed (PHQ-9)	(6) Depressed (PHQ-9)
Variables						
Good HRQoL (1 = good, 0 = else)	−1.482 ***	−1.208 ***	−1.199 ***	−1.206 ***	−1.238 ***	−1.474 ***
	(0.062)	(0.051)	(0.051)	(0.055)	(0.046)	(0.047)
Social networking activity (1 = at least once per month, 0 = no)	−0.185	−0.317 ***	−0.471 ***	−0.507 ***	−0.502 ***	−0.424 ***
	(0.101)	(0.071)	(0.053)	(0.048)	(0.040)	(0.047)
Good HRQoL X social networking activity	0.072	0.125	0.388 ***	0.472 ***	0.533 ***	0.638 ***
	(0.104)	(0.076)	(0.064)	(0.064)	(0.052)	(0.058)
Constant	6.733 ***	7.962 ***	6.213 ***	5.531 ***	5.086 ***	7.676 ***
	(0.678)	(0.705)	(0.550)	(0.457)	(0.444)	(0.552)
Observations	18,874	21,454	30,073	37,770	45,756	48,627
R-squared	0.208	0.186	0.177	0.191	0.191	0.202
Control variables	Yes	Yes	Yes	Yes	Yes	Yes
District FE	Yes	Yes	Yes	Yes	Yes	Yes
Age	>20s	30s	40s	50s	60s	70s +

**Note:** Cluster-robust standard errors by district in parentheses. District here means “si, gun, and gu” in South Korea. PHQ-9 here is a continuous variable. *** *p* < 0.001, ** *p* < 0.01, * *p* < 0.05.

**Table 3:** Results of Stress.

	(1) Stressed (1 = High, 0 = Low)?	(2) Stressed (1 = High, 0 = Low)?	(3) Stressed (1 = high, 0 = Low)?	(4) Stressed (1 = High, 0 = Low)?	(5) Stressed (1 = High, 0 = Low)?	(6) Stressed (1 = High, 0 = Low)?

Variables						
Good HRQoL (1 = good, 0 = else)	−0.138 ***	−0.132 ***	−0.120 ***	−0.106 ***	−0.109 ***	−0.090 ***
	(0.008)	(0.008)	(0.008)	(0.007)	(0.006)	(0.006)
Social networking activity (once per month; 1 = at least once per month, 0 = no)	−0.017	−0.026 *	−0.030 ***	−0.045 ***	−0.052 ***	−0.034 ***
	(0.013)	(0.011)	(0.008)	(0.007)	(0.005)	(0.005)
Good HRQoL X social networking activity	0.022	0.003	0.014	0.023 **	0.040 ***	0.029 ***
	(0.014)	(0.013)	(0.011)	(0.008)	(0.007)	(0.007)
Constant	0.761 ***	0.594 ***	0.480 ***	0.556 ***	0.409 ***	0.422 ***
	(0.095)	(0.101)	(0.087)	(0.069)	(0.050)	(0.050)
Observations	18,874	21,454	30,073	37,770	45,756	48,627
R-squared	0.099	0.082	0.076	0.070	0.071	0.072
Control variables	Yes	Yes	Yes	Yes	Yes	Yes
District FE	Yes	Yes	Yes	Yes	Yes	Yes
Age	>20s	30s	40s	50s	60s	70s +

**Note**: Cluster-robust standard errors by district in parentheses. The results of mental stress show similar patterns found in mental depression models. *** *p* < 0.001, ** *p* < 0.01, * *p* < 0.05.

## Data Availability

The data presented in this study are available upon request from the corresponding author, as the government provides them only through specific requests. Otherwise, the dataset is available by requesting it directly from the Korea Disease Control Agency (see: https://chs.kdca.go.kr/chs/mnl/mnlBoardMain.do (accessed on 1 August 2025)).

## Supplementary Materials

The following supporting information can be downloaded at: https://www.mdpi.com/article/10.3390/jcm14196739/s1, Figure S1: Results of Social Networking on Depression (95% C.I.); Figure S2: Results of Social Networking on Stress (95% C.I.); Table S1: Coding and Summary of Statistics; Stata do-file.
