# Supplementary material for: Quality of Life, Social Networking, and Mental Health: Generational Differentiation and Uniqueness in the Context of South Korea
Source: J Clin Med. 2025 Sep 24;14(19):6739. doi: 10.3390/jcm14196739 (PMC12524798; doi:10.3390/jcm14196739)
Supplement: Supplementary file 1 [file jcm-14-06739-s001.zip › jcm-3855432-supplementary.pdf]

Supplementary Materials

Figure S1. Results of Social Networking on Depression (95% C.I.)

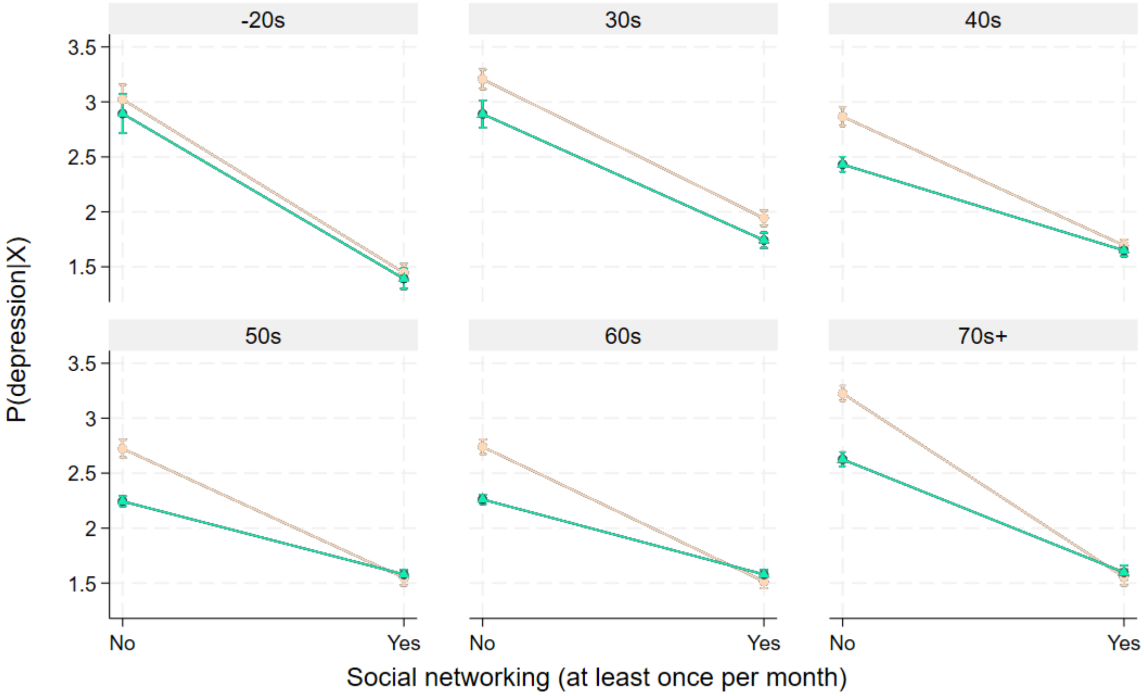

***Note*:** This figure is based on Table 2. As shown, the slope of participation in social networking activities steadily increases with age, consistent with Table 2. The slopes for individuals who participate in social networking are in *green*, and those who do not are shown in *light yellow*.

Figure S2. Results of Social Networking on Stress (95% C.I.)

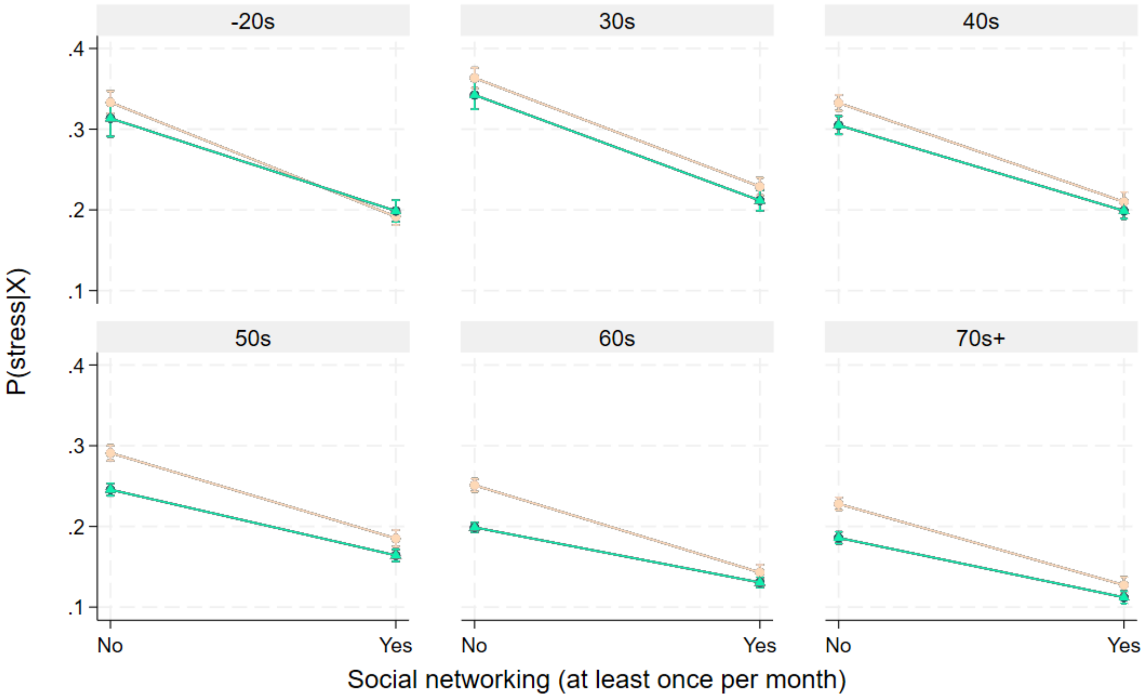

Note: This figure is based on Table 3. As shown, the slope of participation in social networking activities steadily increases with age, consistent with Table 3. The slopes for individuals who participate in social networking are in *green*, and those who do not are shown in *light yellow*.

Supplementary Materials B

Table S1. Coding and Summary of Statistics

VARIABLES	(1) N	(2) Mean	(3) S.D.	(4) Min.	(5) Max.
Dependent Variables					
Depressed (PHQ-9) (=depression0)	203,320	2.298	3.194	0	27
Stressed (1=high, 0=low) (=stress)	203,320	0.224	0.417	0	1
Key Independent Variables of Interest					
Health-related quality of life (1=high, 0=else) (=goodqol)	203,320	0.385	0.487	0	1
Participate in social networking activities at least once per month (1=at least once per month, 0=no) (=enb_05z1)	203,320	0.496	0.500	0	1
Covariates					
Age (=age)	203,320	55.99	17.449	19	106
Sex (1=man, 0=woman) (=sex)	203,320	0.456	0.498	0	1
Neighborhood type code (1=Dong, 2=Eup/Myeon) (=DONG_TY_CODE)	203,320	1.425	0.494	1	2
Housing type code (1=Non-APT, 2=APT) (=HOUSE_TY_CODE)	203,320	1.459	0.498	1	2
Diverse family or not (1=yes, 0=no) (=fma_25z1)	203,320	0.0139	0.117	0	1
Use a safety belt? (1=don't drive, 2=not at all, 3=rarely, 4=sometimes, 5=often, 6=always) (=sfa_02z3)	203,320	3.908	2.399	1	6
High-intensity physical activity in the last week (days) (=pha_04z1)	203,320	0.801	1.677	0	7
Moderate-intensity physical activity in the last week (days) (=pha_07z1)	203,320	1.382	2.166	0	7
Walked more than 10 minutes in a week (days) (=phb_01z1)	203,320	4.263	2.613	0	7
How often did you have your breakfast for the past 1 year (in a week)? (1=5-7 days, 2=3-4days, 3=1-2 days, 4=0 day) (=nua_01z2)	203,320	1.809	1.226	1	4
Height (cm) (=oba_02z1)	202,689	163.9	9.017	50	200
Weight (kg) (=oba_03z1)	203,245	63.93	12.52	20	163
Received influenza vaccine (1=yes, 0=no) (=sca_01z1)	203,320	0.597	0.490	0	1
Had high blood pressure (1=yes, 0=no)? (=hya_04z1)	203,320	0.311	0.463	0	1
Had diabetes (1=yes, 0=no)? (=dia_04z1)	203,320	0.136	0.343	0	1
Haven't received medical service when needed in the past year (1=yes, 2=no, 3=didn't need it)? (=accessmedical)	203,320	2.027	0.360	1	3
Had accident or addicted in the last year (1=yes, 0=no)? (=ira_01z1)	203,320	0.0605	0.238	0	1
Trust your neighbor (1=yes, 0=no)? (=ena_01a1)	203,320	0.709	0.454	0	1
Neighbors help each other for events (1=yes, 0=no)? (=ena_01b1)	203,320	0.485	0.500	0	1
Safe neighborhood (1=yes, 0=no)? (=ena_01c1)	203,320	0.867	0.340	0	1

Good natural environment of the neighborhood (1=yes, 0=no)? (=ena_01d1)	203,320	0.834	0.372	0	1
Neighborhood equipped with good living conditions (1=yes, 0=no)? (=ena_01e1)	203,320	0.857	0.350	0	1
Good access to public transportation (1=yes, 0=no)? (=ena_01f1)	203,320	0.706	0.456	0	1
Satisfactory medical service conditions in the neighborhood (1=yes, 0=no)? (=ena_01g1)	203,320	0.738	0.440	0	1
How often do you contact with your closest family or relative (1=frequently, 0=not so much)? (=enb_01z1)	203,320	0.591	0.492	0	1
How often do you contact with your closest neighbor (1=frequently, 0=not so much)? (=enb_02z1)	203,320	0.505	0.500	0	1
How often do you contact with your closest friend (1=frequently, 0=not so much)? (=enb_03z1)	203,320	0.520	0.500	0	1
Occupation status (1=employer, 2=salaried, 3=working with no salary, 8=unemployed) (=soa_07z1)	203,310	4.019	3.021	1	8
Basic livelihood security beneficiary or not (1=yes, 0=no) (=basicsecurity)	203,320	0.049	0.215	0	1
Ever smoked (1=yes, 0=no)? (=smoked)	203,320	0.387	0.487	0	1
Indirectly smoked (1=yes, 0=no)? (=indirectsmoked)	203,320	0.003	0.053	0	1
Ever drank alcohol (1=yes, 0=no)? (=drank)	203,318	0.834	0.373	0	1
Was on a diet (1=yes, 0=no)? (=diet)	203,319	0.642	0.479	0	1
Good teeth condition (1=yes, 0=no)? (goodteethhealth)	203,320	0.259	0.438	0	1
Ever gambled (1=yes, 0=no)? (=gambled)	203,320	0.280	0.449	0	1
Had a health check-up recently? (Cronbach's alpha=0.77) (=healthcheckup)	203,320	0.697	0.409	0	1
Had a stroke (1=yes, 0=no) (Cronbach's alpha=0.84)? (=stroke)	203,320	0.812	0.302	0	1
Had early signs of heart attack (1=yes, 0=no) (Cronbach's alpha=0.79)? (=earlyperceivhrtatck)	203,320	0.738	0.337	0	1
Had higher education (1=yes, 0=no)? (=higheredu)	203,284	0.403	0.490	0	1
Married (1=yes, 0=no) (=married)	203,306	0.666	0.472	0	1

Note: Please find the summary of statistics with variable names for coding above.

Stata do-file

```
clear all

use "2023 지역건강조사"

replace sex=0 if sex==2
lab define sex 0 "Woman" 1 "Man"
lab value sex sex

lab define dummy 0 "No" 1 "Yes"

lab var CTPRVN_CODE "시도번호"
lab var DONG_TY_CODE "동/읍,면 구분 번호"
lab var HOUSE_TY_CODE "주택유형"
lab var signgu_code "시,군,구 번호"

lab define CTPRVN_CODE 11 "Seoul" 26 "Busan" 27 "Daegu" 28 "Incheon" 29 "Gwangju" 30 "Daejeon" 31 "Ulsan" 36
"Sejong" 41 "Gyeongi" 42 "Gangwon" 43" Chungbuk" 44 "Chungnam" 45 "Jeonbuk" 46 "Jeon-nam" 47 "Gyeongbuk" 48
"Gyeongnam" 50 "Jeju"
lab value CTPRVN_CODE CTPRVN_CODE

lab define DONG_TY_CODE 1 "Dong" 2 "Eup/Myeon"
lab value DONG_TY_CODE DONG_TY_CODE

lab define HOUSE_TY_CODE 1 "Non-APT" 2 "APT"
lab value HOUSE_TY_CODE HOUSE_TY_CODE

*지금까지 기초수급자여부
gen basicsecurity=.
lab var basicsecurity "Basic livelihood security beneficiary or not"
lab value basicsecurity dummy
drop if fma_04z1==7
drop if fma_04z1==9

replace basicsecurity=1 if fma_04z1==1
replace basicsecurity=1 if fma_04z1==2
replace basicsecurity=0 if fma_04z1==3

*Diversity
replace fma_25z1=0 if fma_25z1==2
lab value fma_25z1 dummy
lab var fma_25z1 "다문화 가구여부 (diverse family or not)"

*Quality of life used in this dataset.
lab var qoa_01z1 "Quality of life"
drop if qoa_01z1==7
drop if qoa_01z1==9
lab define qoa_01z1 1 "Very good" 2 "Good" 3 "Neither" 4 "Bad" 5 "Very bad"
lab value qoa_01z1 qoa_01z1

gen goodqol=.
replace goodqol=1 if qoa_01z1==1
replace goodqol=1 if qoa_01z1==2
replace goodqol=0 if qoa_01z1==3
replace goodqol=0 if qoa_01z1==4
replace goodqol=0 if qoa_01z1==5
lab var goodqol "High quality of life (1=high, 0=no)"
```

```
lab define goodqol 0 "No" 1 "High"
lab value goodqol goodqol




*Ever smoked so far?
drop if smf_01z1==7
drop if smf_01z1==9

gen smoked=.
replace smoked=1 if smf_01z1==1
replace smoked=0 if smf_01z1==2
lab var smf_01z1 "Ever smoked (1=yes, 0=no)?"
lab value smf_01z1 dummy


*Indirectly smoked
gen indirectsmoked=.
replace indirectsmoked=1 if smc_08z2==1
replace indirectsmoked=1 if smc_09z2==1
replace indirectsmoked=1 if smc_10z2==1
replace indirectsmoked=1 if smc_07z3==1
replace indirectsmoked=1 if smc_07z4==1

replace indirectsmoked=0 if smc_08z2==2
replace indirectsmoked=0 if smc_09z2==2
replace indirectsmoked=0 if smc_10z2==2
replace indirectsmoked=0 if smc_07z3==2
replace indirectsmoked=0 if smc_07z4==2

lab var indirectsmoked "Indirectly smoked (1=yes, 0=no)"
lab value indirectsmoked dummy



*Use of safety belt

lab define sfa_02z3 1 "Don't drive" 2 "Never" 3 "Rarely" 4 "Sometimes" 5 "Usually" 6 "Always"
drop if sfa_02z3==7
drop if sfa_02z3==9





*Ever drank for the last 1 year?
gen drank=.
replace drank=1 if dra_01z1==1
replace drank=0 if dra_01z1==2
lab value drank dummy
lab var drank "Ever drank for the last 1 year?"



*Excercise: How many days in a week?

drop if pha_04z1==77
drop if pha_04z1==99
*High-level exercise

drop if pha_07z1==77
drop if pha_07z1==99
*mid-level exercise

drop if phb_01z1==77
drop if phb_01z1==99
*walking



*How many days do you eat breakfast each week (during the past 1 year)?

drop if nua_01z2==7
drop if nua_01z2==9


*Height and weight

lab var oba_02z1 "Height (cm)"
lab var oba_03z1 "Weight (kg)"
```

```
drop if oba_02z1==77777
drop if oba_03z1==99999
drop if oba_02z1==77777
drop if oba_03z1==99999

replace oba_02z1=. if oba_02z1==77777
replace oba_02z1=. if oba_02z1==99999

replace oba_03z1=. if oba_03z1==77777
replace oba_03z1=. if oba_03z1==99999



*Was on a diet?

gen diet=.
replace diet=1 if obb_01z1==1
replace diet=1 if obb_01z1==2
replace diet=1 if obb_01z1==3
replace diet=0 if obb_01z1==4
lab var diet "Was on a diet (1=yes, 0=no)?"
lab value diet dummy


*Teeth health condition

gen goodteethhealth=.
replace goodteethhealth=1 if ora_01z1==1
replace goodteethhealth=1 if ora_01z1==2
replace goodteethhealth=0 if ora_01z1==3
replace goodteethhealth=0 if ora_01z1==4
replace goodteethhealth=0 if ora_01z1==5

lab var goodteethhealth "Good teeth condition (1=yes, 0=no)?"
lab define goodteethhealth 0 "No" 1 "Good"
lab value goodteethhealth goodteethhealth


*stress
drop if mta_01z1==7
drop if mta_01z1==9

gen stress=.
replace stress=1 if mta_01z1==1
replace stress=1 if mta_01z1==2
replace stress=0 if mta_01z1==3
replace stress=0 if mta_01z1==4
lab value stress dummy
lab var stress "Stressed (1=yes, 0=no)?"


*Drepression
drop if mtb_07a1==7
drop if mtb_07a1==9

drop if mtb_07b1==7
drop if mtb_07b1==9

drop if mtb_07c1==7
drop if mtb_07c1==9

drop if mtb_07d1==7
drop if mtb_07d1==9

drop if mtb_07e1==7
drop if mtb_07e1==9


drop if mtb_07f1==7
drop if mtb_07f1==9

drop if mtb_07g1==7
drop if mtb_07g1==9

drop if mtb_07h1==7
drop if mtb_07h1==9

drop if mtb_07i1==7
drop if mtb_07i1==9
```

```
gen depression0 = mtb_07a1+mtb_07b1+mtb_07c1+mtb_07d1+mtb_07e1+mtb_07f1+mtb_07g1+mtb_07h1+mtb_07i1-9

sum depression0


alpha mtb_07a1 mtb_07b1 mtb_07c1 mtb_07d1 mtb_07e1 mtb_07f1 mtb_07g1 mtb_07h1 mtb_07i1, gen(depressionalpha)
item label


gen lowdepression =.
replace lowdepression=1 if depression0>=5
replace lowdepression=0 if depression0<5
lab var lowdepression "Low depression (1=yes or higher, 0=no)"

gen depression=.
replace depression=1 if depression0>=10
replace depression=0 if depression0<10

lab var depression "Depression (1=yes, 0=no)"




*Gambled

gen gambled=.
replace gambled=0 if mti_01z1==1
replace gambled=1 if mti_01z1==2
replace gambled=1 if mti_01z1==3
replace gambled=1 if mti_01z1==4
replace gambled=1 if mti_01z1==5
replace gambled=1 if mti_01z1==6

lab var gambled "Ever gambled (1=yes, 0=no)?"
lab value gambled dummy

*received influenza vaccine

replace sca_01z1=0 if sca_01z1==2
drop if sca_01z1==7
drop if sca_01z1==9
lab value sca_01z1 dummy


*Health check-up for the last 2 years.

drop if scb_01z1==7
drop if scb_01z1==9

drop if scc_01z1==7
drop if scc_01z1==9

gen noncancerhealthcheckup=.
replace noncancerhealthcheckup=1 if scb_01z1==1
replace noncancerhealthcheckup=0 if scb_01z1==2
lab var noncancerhealthcheckup "Non-cancer health check-up"

gen cancerhealthcheckup=.
replace cancerhealthcheckup=1 if scc_01z1==1
replace cancerhealthcheckup=0 if scc_01z1==2
lab var cancerhealthcheckup "Cancer health check-up"

alpha cancerhealthcheckup noncancerhealthcheckup, gen(healthcheckup) item label
lab var healthcheckup "Health check-up in the last 2 years (alpha=0.77)?"



*Stroke

replace cva_11z2=0 if cva_11z2==2
drop if cva_11z2==7
drop if cva_11z2==9

replace cva_12z1=0 if cva_12z1==2
drop if cva_12z1==7
drop if cva_12z1==9

replace cva_14z2=0 if cva_14z2==2
drop if cva_14z2==7
drop if cva_14z2==9
```

```
replace cva_16z2=0 if cva_16z2==2
drop if cva_16z2==7
drop if cva_16z2==9

replace cva_17z1=0 if cva_17z1==2
drop if cva_17z1==7
drop if cva_17z1==9

alpha cva_11z2 cva_12z1 cva_14z2 cva_16z2 cva_17z1, gen (stroke) item label
lab var stroke "Stroke (alpha=0.84)?"



* (Myocardial infarction)


replace mya_10z2=0 if mya_10z2==2
drop if mya_10z2==7
drop if mya_10z2==9

replace mya_11z2=0 if mya_11z2==2
drop if mya_11z2==7
drop if mya_11z2==9

replace mya_12z2=0 if mya_12z2==2
drop if mya_12z2==7
drop if mya_12z2==9

replace mya_14z1=0 if mya_14z1==2
drop if mya_14z1==7
drop if mya_14z1==9

replace mya_15z1=0 if mya_15z1==2
drop if mya_15z1==7
drop if mya_15z1==9

alpha mya_10z2 mya_11z2 mya_11z2 mya_14z1 mya_15z1, gen(earlyperceivhrtatck) item label
lab var earlyperceivhrtatck "Early recognition of heart attack (alpha=0.79)"

*Ever had a high blood pressure

replace hya_04z1=0 if hya_04z1==2
drop if hya_04z1==7
drop if hya_04z1==9

*Ever had diabetes?

replace dia_04z1=0 if dia_04z1==2
drop if dia_04z1==7
drop if dia_04z1==9

*Access to medical service

lab define accessmedical 1 "Could not access" 2 "Had access" 3 "Didn't need medical service at all"
drop if sra_01z3==7
drop if sra_01z3==9


*Accident or addiction
drop if ira_01z1==7
drop if ira_01z1==9
replace ira_01z1=0 if ira_01z1==2
lab value ira_01z1 dummy


*Socio-physical environment

replace ena_01a1=0 if ena_01a1==2
drop if ena_01a1==7
drop if ena_01a1==9

replace ena_01b1=0 if ena_01b1==2
drop if ena_01b1==7
drop if ena_01b1==9

replace ena_01c1=0 if ena_01c1==2
drop if ena_01c1==7
drop if ena_01c1==9

replace ena_01d1=0 if ena_01d1==2
```

```
drop if ena_01d1==7
drop if ena_01d1==9

replace ena_01e1=0 if ena_01e1==2
drop if ena_01e1==7
drop if ena_01e1==9

replace ena_01f1=0 if ena_01f1==2
drop if ena_01f1==7
drop if ena_01f1==9

replace ena_01g1=0 if ena_01g1==2
drop if ena_01g1==7
drop if ena_01g1==9


*Control variable: Contact frequency with others
lab define highlow 0 "Low" 1 "High"

drop if enb_01z1==7
drop if enb_01z1==9
replace enb_01z1=0 if enb_01z1==1
replace enb_01z1=0 if enb_01z1==2
replace enb_01z1=0 if enb_01z1==3
replace enb_01z1=1 if enb_01z1==4
replace enb_01z1=1 if enb_01z1==5
replace enb_01z1=1 if enb_01z1==6
lab value enb_01z1 highlow

drop if enb_02z1==7
drop if enb_02z1==9
replace enb_02z1=0 if enb_02z1==1
replace enb_02z1=0 if enb_02z1==2
replace enb_02z1=0 if enb_02z1==3
replace enb_02z1=1 if enb_02z1==4
replace enb_02z1=1 if enb_02z1==5
replace enb_02z1=1 if enb_02z1==6
lab value enb_02z1 highlow


drop if enb_03z1==7
drop if enb_03z1==9
replace enb_03z1=0 if enb_03z1==1
replace enb_03z1=0 if enb_03z1==2
replace enb_03z1=0 if enb_03z1==3
replace enb_03z1=1 if enb_03z1==4
replace enb_03z1=1 if enb_03z1==5
replace enb_03z1=1 if enb_03z1==6
lab value enb_03z1 highlow




replace enb_04z1=0 if enb_04z1==2
drop if enb_04z1==7
drop if enb_04z1==9

replace enb_05z1=0 if enb_05z1==2
drop if enb_05z1==7
drop if enb_05z1==9

lab var enb_05z1 "Social networking activity (at least once per month) (1=yes, 0=no)"
lab define enb_05z1 0 "No" 1 "At least once per month"
lab value enb_05z1 enb_05z1


replace enb_06z1=0 if enb_06z1==2
drop if enb_06z1==7
drop if enb_06z1==9

replace enb_07z1=0 if enb_07z1==2
drop if enb_07z1==7
drop if enb_07z1==9




*Age group
gen agegroup=.
replace agegroup=1 if age<20
```

```
replace agegroup=2 if 20<=age & age<30
replace agegroup=3 if 30<=age & age<40
replace agegroup=4 if 40<=age & age<50
replace agegroup=5 if 50<=age & age<60
replace agegroup=6 if 60<=age & age<70
replace agegroup=7 if 70<=age

lab define agegroup 1 "20s and below" 2 "20s" 3 "30s" 4 "40s" 5 "50s" 6 "60s" 7 "70s and above"
lab value agegroup agegroup
lab var agegroup "Age group (10-year bracket)"

codebook agegroup

*Higher education

gen higheredu=.
replace higheredu=1 if sob_01z1==8
replace higheredu=1 if sob_01z1==7
replace higheredu=1 if sob_01z1==6
replace higheredu=0 if sob_01z1==5
replace higheredu=0 if sob_01z1==4
replace higheredu=0 if sob_01z1==3
replace higheredu=0 if sob_01z1==2
replace higheredu=0 if sob_01z1==1
lab var higheredu "Received higher education (1=yes, 0=no)?"
lab value higheredu dummy



gen interaction=goodqol*enb_05z1




*Occupation
replace soa_07z1=. if soa_07z1==7
replace soa_07z1=. if soa_07z1==9
lab define soa_07z1 1 "Employer or self-employed" 2 "Salary" 3 "No salary or income" 8 "Unemployed"





*Married status

gen married=.
replace married=1 if sod_02z3==1
replace married=1 if sod_02z3==2
replace married=0 if sod_02z3==3
replace married=0 if sod_02z3==4
replace married=0 if sod_02z3==5
lab var married "Married status (1=married, 0=not married)"






****************************using PHQ-9**********************



areg depression0 i.goodqol##i.enb_05z1 i.sex  i.HOUSE_TY_CODE i.DONG_TY_CODE i.HOUSE_TY_CODE  i.basicsecurity
i.fma_25z1 i.smoked i.indirectsmoked i.sfa_02z3 i.drank i.pha_04z1 i.pha_07z1 i.phb_01z1 nua_01z2 oba_02z1
oba_03z1 i.diet goodteethhealth  i.gambled i.sca_01z1 healthcheckup stroke earlyperceivhrtatck i.hya_04z1
i.dia_04z1 i.sra_01z3 i.ira_01z1 i.ena_01a1 i.ena_01b1 i.ena_01c1 i.ena_01d1 i.ena_01e1 i.ena_01f1 i.ena_01g1
i.enb_01z1 i.enb_02z1 i.enb_03z1 i.higheredu  i.soa_07z1 i.married if agegroup<=2 , absorb(signgu_code)
vce(cluster signgu_code)
outreg2 using "Depressionresults.xls", paren(se) aster(coef) alpha(0.001, 0.01, 0.05) dec(3) label append
addtext(Control variables, Yes, District FE, Yes, Age, - 20s) keep(i.goodqol##i.enb_05z1)



areg depression0 i.goodqol##i.enb_05z1 i.sex  i.HOUSE_TY_CODE i.DONG_TY_CODE i.HOUSE_TY_CODE  i.basicsecurity
i.fma_25z1 i.smoked i.indirectsmoked i.sfa_02z3 i.drank i.pha_04z1 i.pha_07z1 i.phb_01z1 nua_01z2 oba_02z1
oba_03z1 i.diet goodteethhealth  i.gambled i.sca_01z1 healthcheckup stroke earlyperceivhrtatck i.hya_04z1
i.dia_04z1 i.sra_01z3 i.ira_01z1 i.ena_01a1 i.ena_01b1 i.ena_01c1 i.ena_01d1 i.ena_01e1 i.ena_01f1 i.ena_01g1
i.enb_01z1 i.enb_02z1 i.enb_03z1 i.higheredu  i.soa_07z1 i.married if agegroup==3 , absorb(signgu_code)
vce(cluster signgu_code)
outreg2 using "Depressionresults.xls", paren(se) aster(coef) alpha(0.001, 0.01, 0.05) dec(3) label append
addtext(Control variables, Yes, District FE, Yes, Age, 30s) keep(i.goodqol##i.enb_05z1)
```

```
areg depression0 i.goodqol##i.enb_05z1 i.sex  i.HOUSE_TY_CODE i.DONG_TY_CODE i.HOUSE_TY_CODE  i.basicsecurity
i.fma_25z1 i.smoked i.indirectsmoked i.sfa_02z3 i.drank i.pha_04z1 i.pha_07z1 i.phb_01z1 nua_01z2 oba_02z1
oba_03z1 i.diet goodteethhealth  i.gambled i.sca_01z1 healthcheckup stroke earlyperceivhrtatck i.hya_04z1
i.dia_04z1 i.sra_01z3 i.ira_01z1 i.ena_01a1 i.ena_01b1 i.ena_01c1 i.ena_01d1 i.ena_01e1 i.ena_01f1 i.ena_01g1
i.enb_01z1 i.enb_02z1 i.enb_03z1 i.higheredu  i.soa_07z1 i.married if agegroup==4 , absorb(signgu_code)
vce(cluster signgu_code)
outreg2 using "Depressionresults.xls", paren(se) aster(coef) alpha(0.001, 0.01, 0.05) dec(3) label append
addtext(Control variables, Yes, District FE, Yes, Age, 40s) keep(i.goodqol##i.enb_05z1)
```

```
areg depression0 i.goodqol##i.enb_05z1 i.sex  i.HOUSE_TY_CODE i.DONG_TY_CODE i.HOUSE_TY_CODE  i.basicsecurity
i.fma_25z1 i.smoked i.indirectsmoked i.sfa_02z3 i.drank i.pha_04z1 i.pha_07z1 i.phb_01z1 nua_01z2 oba_02z1
oba_03z1 i.diet goodteethhealth  i.gambled i.sca_01z1 healthcheckup stroke earlyperceivhrtatck i.hya_04z1
i.dia_04z1 i.sra_01z3 i.ira_01z1 i.ena_01a1 i.ena_01b1 i.ena_01c1 i.ena_01d1 i.ena_01e1 i.ena_01f1 i.ena_01g1
i.enb_01z1 i.enb_02z1 i.enb_03z1 i.higheredu  i.soa_07z1 i.married if agegroup==5 , absorb(signgu_code)
vce(cluster signgu_code)
outreg2 using "Depressionresults.xls", paren(se) aster(coef) alpha(0.001, 0.01, 0.05) dec(3) label append
addtext(Control variables, Yes, District FE, Yes, Age, 50s) keep(i.goodqol##i.enb_05z1)
```

```
areg depression0 i.goodqol##i.enb_05z1 i.sex  i.HOUSE_TY_CODE i.DONG_TY_CODE i.HOUSE_TY_CODE  i.basicsecurity
i.fma_25z1 i.smoked i.indirectsmoked i.sfa_02z3 i.drank i.pha_04z1 i.pha_07z1 i.phb_01z1 nua_01z2 oba_02z1
oba_03z1 i.diet goodteethhealth  i.gambled i.sca_01z1 healthcheckup stroke earlyperceivhrtatck i.hya_04z1
i.dia_04z1 i.sra_01z3 i.ira_01z1 i.ena_01a1 i.ena_01b1 i.ena_01c1 i.ena_01d1 i.ena_01e1 i.ena_01f1 i.ena_01g1
i.enb_01z1 i.enb_02z1 i.enb_03z1 i.higheredu  i.soa_07z1 i.married if agegroup==6 , absorb(signgu_code)
vce(cluster signgu_code)
outreg2 using "Depressionresults.xls", paren(se) aster(coef) alpha(0.001, 0.01, 0.05) dec(3) label append
addtext(Control variables, Yes, District FE, Yes, Age, 60s) keep(i.goodqol##i.enb_05z1)
```

```
areg depression0 i.goodqol##i.enb_05z1 i.sex  i.HOUSE_TY_CODE i.DONG_TY_CODE i.HOUSE_TY_CODE  i.basicsecurity
i.fma_25z1 i.smoked i.indirectsmoked i.sfa_02z3 i.drank i.pha_04z1 i.pha_07z1 i.phb_01z1 nua_01z2 oba_02z1
oba_03z1 i.diet goodteethhealth  i.gambled i.sca_01z1 healthcheckup stroke earlyperceivhrtatck i.hya_04z1
i.dia_04z1 i.sra_01z3 i.ira_01z1 i.ena_01a1 i.ena_01b1 i.ena_01c1 i.ena_01d1 i.ena_01e1 i.ena_01f1 i.ena_01g1
i.enb_01z1 i.enb_02z1 i.enb_03z1 i.higheredu  i.soa_07z1 i.married if agegroup==7 , absorb(signgu_code)
vce(cluster signgu_code)
outreg2 using "Depressionresults.xls", paren(se) aster(coef) alpha(0.001, 0.01, 0.05) dec(3) label append
addtext(Control variables, Yes, District FE, Yes, Age, 70s -) keep(i.goodqol##i.enb_05z1)
ytitle(P(depression|X))
```

```
areg stress i.goodqol##i.enb_05z1 i.sex  i.HOUSE_TY_CODE i.DONG_TY_CODE i.HOUSE_TY_CODE  i.basicsecurity
i.fma_25z1 i.smoked i.indirectsmoked i.sfa_02z3 i.drank i.pha_04z1 i.pha_07z1 i.phb_01z1 nua_01z2 oba_02z1
oba_03z1 i.diet goodteethhealth  i.gambled i.sca_01z1 healthcheckup stroke earlyperceivhrtatck i.hya_04z1
i.dia_04z1 i.sra_01z3 i.ira_01z1 i.ena_01a1 i.ena_01b1 i.ena_01c1 i.ena_01d1 i.ena_01e1 i.ena_01f1 i.ena_01g1
i.enb_01z1 i.enb_02z1 i.enb_03z1 i.higheredu  i.soa_07z1 i.married if agegroup<=2 , absorb(signgu_code)
vce(cluster signgu_code)
outreg2 using "Stressresults.xls", paren(se) aster(coef) alpha(0.001, 0.01, 0.05) dec(3) label append
addtext(Control variables, Yes, District FE, Yes, Age, - 20s) keep(i.goodqol##i.enb_05z1)
```

```
areg stress i.goodqol##i.enb_05z1 i.sex  i.HOUSE_TY_CODE i.DONG_TY_CODE i.HOUSE_TY_CODE  i.basicsecurity
i.fma_25z1 i.smoked i.indirectsmoked i.sfa_02z3 i.drank i.pha_04z1 i.pha_07z1 i.phb_01z1 nua_01z2 oba_02z1
oba_03z1 i.diet goodteethhealth  i.gambled i.sca_01z1 healthcheckup stroke earlyperceivhrtatck i.hya_04z1
i.dia_04z1 i.sra_01z3 i.ira_01z1 i.ena_01a1 i.ena_01b1 i.ena_01c1 i.ena_01d1 i.ena_01e1 i.ena_01f1 i.ena_01g1
i.enb_01z1 i.enb_02z1 i.enb_03z1 i.higheredu  i.soa_07z1 i.married if agegroup==3 , absorb(signgu_code)
vce(cluster signgu_code)
outreg2 using "Stressresults.xls", paren(se) aster(coef) alpha(0.001, 0.01, 0.05) dec(3) label append
addtext(Control variables, Yes, District FE, Yes, Age, 30s) keep(i.goodqol##i.enb_05z1)
```

```
areg stress i.goodqol##i.enb_05z1 i.sex  i.HOUSE_TY_CODE i.DONG_TY_CODE i.HOUSE_TY_CODE  i.basicsecurity
i.fma_25z1 i.smoked i.indirectsmoked i.sfa_02z3 i.drank i.pha_04z1 i.pha_07z1 i.phb_01z1 nua_01z2 oba_02z1
oba_03z1 i.diet goodteethhealth  i.gambled i.sca_01z1 healthcheckup stroke earlyperceivhrtatck i.hya_04z1
i.dia_04z1 i.sra_01z3 i.ira_01z1 i.ena_01a1 i.ena_01b1 i.ena_01c1 i.ena_01d1 i.ena_01e1 i.ena_01f1 i.ena_01g1
i.enb_01z1 i.enb_02z1 i.enb_03z1 i.higheredu  i.soa_07z1 i.married if agegroup==4 , absorb(signgu_code)
vce(cluster signgu_code)
outreg2 using "Stressresults.xls", paren(se) aster(coef) alpha(0.001, 0.01, 0.05) dec(3) label append
addtext(Control variables, Yes, District FE, Yes, Age, 40s) keep(i.goodqol##i.enb_05z1)
```

```
areg stress i.goodqol##i.enb_05z1 i.sex  i.HOUSE_TY_CODE i.DONG_TY_CODE i.HOUSE_TY_CODE  i.basicsecurity
i.fma_25z1 i.smoked i.indirectsmoked i.sfa_02z3 i.drank i.pha_04z1 i.pha_07z1 i.phb_01z1 nua_01z2 oba_02z1
oba_03z1 i.diet goodteethhealth  i.gambled i.sca_01z1 healthcheckup stroke earlyperceivhrtatck i.hya_04z1
i.dia_04z1 i.sra_01z3 i.ira_01z1 i.ena_01a1 i.ena_01b1 i.ena_01c1 i.ena_01d1 i.ena_01e1 i.ena_01f1 i.ena_01g1
i.enb_01z1 i.enb_02z1 i.enb_03z1 i.higheredu  i.soa_07z1 i.married if agegroup==5 , absorb(signgu_code)
vce(cluster signgu_code)
outreg2 using "Stressresults.xls", paren(se) aster(coef) alpha(0.001, 0.01, 0.05) dec(3) label append
addtext(Control variables, Yes, District FE, Yes, Age, 50s) keep(i.goodqol##i.enb_05z1)


areg stress i.goodqol##i.enb_05z1 i.sex  i.HOUSE_TY_CODE i.DONG_TY_CODE i.HOUSE_TY_CODE  i.basicsecurity
i.fma_25z1 i.smoked i.indirectsmoked i.sfa_02z3 i.drank i.pha_04z1 i.pha_07z1 i.phb_01z1 nua_01z2 oba_02z1
oba_03z1 i.diet goodteethhealth  i.gambled i.sca_01z1 healthcheckup stroke earlyperceivhrtatck i.hya_04z1
i.dia_04z1 i.sra_01z3 i.ira_01z1 i.ena_01a1 i.ena_01b1 i.ena_01c1 i.ena_01d1 i.ena_01e1 i.ena_01f1 i.ena_01g1
i.enb_01z1 i.enb_02z1 i.enb_03z1 i.higheredu  i.soa_07z1 i.married if agegroup==6 , absorb(signgu_code)
vce(cluster signgu_code)
outreg2 using "Stressresults.xls", paren(se) aster(coef) alpha(0.001, 0.01, 0.05) dec(3) label append
addtext(Control variables, Yes, District FE, Yes, Age, 60s) keep(i.goodqol##i.enb_05z1)


areg stress i.goodqol##i.enb_05z1 i.sex  i.HOUSE_TY_CODE i.DONG_TY_CODE i.HOUSE_TY_CODE  i.basicsecurity
i.fma_25z1 i.smoked i.indirectsmoked i.sfa_02z3 i.drank i.pha_04z1 i.pha_07z1 i.phb_01z1 nua_01z2 oba_02z1
oba_03z1 i.diet goodteethhealth  i.gambled i.sca_01z1 healthcheckup stroke earlyperceivhrtatck i.hya_04z1
i.dia_04z1 i.sra_01z3 i.ira_01z1 i.ena_01a1 i.ena_01b1 i.ena_01c1 i.ena_01d1 i.ena_01e1 i.ena_01f1 i.ena_01g1
i.enb_01z1 i.enb_02z1 i.enb_03z1 i.higheredu  i.soa_07z1 i.married if agegroup==7 , absorb(signgu_code)
vce(cluster signgu_code)
outreg2 using "Stressresults.xls", paren(se) aster(coef) alpha(0.001, 0.01, 0.05) dec(3) label append
addtext(Control variables, Yes, District FE, Yes, Age, 70s -) keep(i.goodqol##i.enb_05z1)




quietly: areg depression0 i.goodqol##i.enb_05z1 i.sex  i.HOUSE_TY_CODE i.DONG_TY_CODE i.HOUSE_TY_CODE
i.basicsecurity  i.fma_25z1 i.smoked i.indirectsmoked i.sfa_02z3 i.drank i.pha_04z1 i.pha_07z1 i.phb_01z1
nua_01z2 oba_02z1 oba_03z1 i.diet goodteethhealth  i.gambled i.sca_01z1 healthcheckup stroke
earlyperceivhrtatck i.hya_04z1 i.dia_04z1 i.sra_01z3 i.ira_01z1 i.ena_01a1 i.ena_01b1 i.ena_01c1 i.ena_01d1
i.ena_01e1 i.ena_01f1 i.ena_01g1 i.enb_01z1 i.enb_02z1 i.enb_03z1 i.higheredu  i.soa_07z1 i.married  ,
absorb(signgu_code)
est store yes
quietly: areg depression0 i.goodqol i.enb_05z1 i.sex  i.HOUSE_TY_CODE i.DONG_TY_CODE i.HOUSE_TY_CODE
i.basicsecurity  i.fma_25z1 i.smoked i.indirectsmoked i.sfa_02z3 i.drank i.pha_04z1 i.pha_07z1 i.phb_01z1
nua_01z2 oba_02z1 oba_03z1 i.diet goodteethhealth  i.gambled i.sca_01z1 healthcheckup stroke
earlyperceivhrtatck i.hya_04z1 i.dia_04z1 i.sra_01z3 i.ira_01z1 i.ena_01a1 i.ena_01b1 i.ena_01c1 i.ena_01d1
i.ena_01e1 i.ena_01f1 i.ena_01g1 i.enb_01z1 i.enb_02z1 i.enb_03z1 i.higheredu  i.soa_07z1 i.married  ,
absorb(signgu_code)
est store no
lrtest yes no

quietly: areg stress i.goodqol##i.enb_05z1 i.sex  i.HOUSE_TY_CODE i.DONG_TY_CODE i.HOUSE_TY_CODE
i.basicsecurity  i.fma_25z1 i.smoked i.indirectsmoked i.sfa_02z3 i.drank i.pha_04z1 i.pha_07z1 i.phb_01z1
nua_01z2 oba_02z1 oba_03z1 i.diet goodteethhealth  i.gambled i.sca_01z1 healthcheckup stroke
earlyperceivhrtatck i.hya_04z1 i.dia_04z1 i.sra_01z3 i.ira_01z1 i.ena_01a1 i.ena_01b1 i.ena_01c1 i.ena_01d1
i.ena_01e1 i.ena_01f1 i.ena_01g1 i.enb_01z1 i.enb_02z1 i.enb_03z1 i.higheredu  i.soa_07z1 i.married  ,
absorb(signgu_code)
est store yes
quietly: areg stress i.goodqol i.enb_05z1 i.sex  i.HOUSE_TY_CODE i.DONG_TY_CODE i.HOUSE_TY_CODE
i.basicsecurity  i.fma_25z1 i.smoked i.indirectsmoked i.sfa_02z3 i.drank i.pha_04z1 i.pha_07z1 i.phb_01z1
nua_01z2 oba_02z1 oba_03z1 i.diet goodteethhealth  i.gambled i.sca_01z1 healthcheckup stroke
earlyperceivhrtatck i.hya_04z1 i.dia_04z1 i.sra_01z3 i.ira_01z1 i.ena_01a1 i.ena_01b1 i.ena_01c1 i.ena_01d1
i.ena_01e1 i.ena_01f1 i.ena_01g1 i.enb_01z1 i.enb_02z1 i.enb_03z1 i.higheredu  i.soa_07z1 i.married  ,
absorb(signgu_code)
est store no
lrtest yes no




/*

sum depression0 stress goodqol enb_05z1 age sex  HOUSE_TY_CODE DONG_TY_CODE HOUSE_TY_CODE  basicsecurity
fma_25z1 smoked indirectsmoked sfa_02z3 drank pha_04z1 pha_07z1 phb_01z1 nua_01z2 oba_02z1 oba_03z1 diet
goodteethhealth  gambled sca_01z1 healthcheckup stroke earlyperceivhrtatck hya_04z1 dia_04z1 sra_01z3 ira_01z1
ena_01a1 ena_01b1 ena_01c1 ena_01d1 ena_01e1 ena_01f1 ena_01g1 enb_01z1 enb_02z1 enb_03z1 higheredu  soa_07z1
married

outreg2 using "summary_table.xls", word replace sum(log) keep (depression0 stress goodqol enb_05z1 age sex
HOUSE_TY_CODE DONG_TY_CODE HOUSE_TY_CODE  basicsecurity  fma_25z1 smoked indirectsmoked sfa_02z3 drank pha_04z1
pha_07z1 phb_01z1 nua_01z2 oba_02z1 oba_03z1 diet goodteethhealth  gambled sca_01z1 healthcheckup stroke
```

```
earlyperceivhrtatck hya_04z1 dia_04z1 sra_01z3 ira_01z1 ena_01a1 ena_01b1 ena_01c1 ena_01d1 ena_01e1 ena_01f1
ena_01g1 enb_01z1 enb_02z1 enb_03z1 higheredu  soa_07z1 married)
*/

***********Depression


teffects ipw (depression0) (enb_05z1 i.sex  i.HOUSE_TY_CODE i.DONG_TY_CODE i.HOUSE_TY_CODE  i.basicsecurity
i.fma_25z1 i.smoked i.indirectsmoked i.sfa_02z3 i.drank i.pha_04z1 i.pha_07z1 i.phb_01z1 nua_01z2 oba_02z1
oba_03z1 i.diet goodteethhealth  i.gambled i.sca_01z1 healthcheckup stroke earlyperceivhrtatck i.hya_04z1
i.dia_04z1 i.sra_01z3 i.ira_01z1 i.ena_01a1 i.ena_01b1 i.ena_01c1 i.ena_01d1 i.ena_01e1 i.ena_01f1 i.ena_01g1
i.enb_01z1 i.enb_02z1 i.enb_03z1 i.higheredu  i.soa_07z1 i.married i.CTPRVN_CODE) if agegroup<=2 & goodqol==1,
vce(robust)
tebalance sum
est store highqol2

teffects ipw (depression0) (enb_05z1 i.sex  i.HOUSE_TY_CODE i.DONG_TY_CODE i.HOUSE_TY_CODE  i.basicsecurity
i.fma_25z1 i.smoked i.indirectsmoked i.sfa_02z3 i.drank i.pha_04z1 i.pha_07z1 i.phb_01z1 nua_01z2 oba_02z1
oba_03z1 i.diet goodteethhealth  i.gambled i.sca_01z1 healthcheckup stroke earlyperceivhrtatck i.hya_04z1
i.dia_04z1 i.sra_01z3 i.ira_01z1 i.ena_01a1 i.ena_01b1 i.ena_01c1 i.ena_01d1 i.ena_01e1 i.ena_01f1 i.ena_01g1
i.enb_01z1 i.enb_02z1 i.enb_03z1 i.higheredu  i.soa_07z1 i.married i.CTPRVN_CODE) if agegroup<=2 & goodqol==0,
vce(robust)
tebalance sum
est store lowqol2


coefplot lowqol2 highqol2, yline(0) level(95) vertical ytitle(P(depression|X))




teffects ipw (depression0) (enb_05z1 i.sex  i.HOUSE_TY_CODE i.DONG_TY_CODE i.HOUSE_TY_CODE  i.basicsecurity
i.fma_25z1 i.smoked i.indirectsmoked i.sfa_02z3 i.drank i.pha_04z1 i.pha_07z1 i.phb_01z1 nua_01z2 oba_02z1
oba_03z1 i.diet goodteethhealth  i.gambled i.sca_01z1 healthcheckup stroke earlyperceivhrtatck i.hya_04z1
i.dia_04z1 i.sra_01z3 i.ira_01z1 i.ena_01a1 i.ena_01b1 i.ena_01c1 i.ena_01d1 i.ena_01e1 i.ena_01f1 i.ena_01g1
i.enb_01z1 i.enb_02z1 i.enb_03z1 i.higheredu  i.soa_07z1 i.married i.CTPRVN_CODE) if agegroup==3 & goodqol==1,
vce(robust)
tebalance sum
est store highqol3

teffects ipw (depression0) (enb_05z1 i.sex  i.HOUSE_TY_CODE i.DONG_TY_CODE i.HOUSE_TY_CODE  i.basicsecurity
i.fma_25z1 i.smoked i.indirectsmoked i.sfa_02z3 i.drank i.pha_04z1 i.pha_07z1 i.phb_01z1 nua_01z2 oba_02z1
oba_03z1 i.diet goodteethhealth  i.gambled i.sca_01z1 healthcheckup stroke earlyperceivhrtatck i.hya_04z1
i.dia_04z1 i.sra_01z3 i.ira_01z1 i.ena_01a1 i.ena_01b1 i.ena_01c1 i.ena_01d1 i.ena_01e1 i.ena_01f1 i.ena_01g1
i.enb_01z1 i.enb_02z1 i.enb_03z1 i.higheredu  i.soa_07z1 i.married i.CTPRVN_CODE) if agegroup==3 & goodqol==0,
vce(robust)
tebalance sum
est store lowqol3


coefplot lowqol3 highqol3, yline(0) level(95) vertical ytitle(P(depression|X))



teffects ipw (depression0) (enb_05z1 i.sex  i.HOUSE_TY_CODE i.DONG_TY_CODE i.HOUSE_TY_CODE  i.basicsecurity
i.fma_25z1 i.smoked i.indirectsmoked i.sfa_02z3 i.drank i.pha_04z1 i.pha_07z1 i.phb_01z1 nua_01z2 oba_02z1
oba_03z1 i.diet goodteethhealth  i.gambled i.sca_01z1 healthcheckup stroke earlyperceivhrtatck i.hya_04z1
i.dia_04z1 i.sra_01z3 i.ira_01z1 i.ena_01a1 i.ena_01b1 i.ena_01c1 i.ena_01d1 i.ena_01e1 i.ena_01f1 i.ena_01g1
i.enb_01z1 i.enb_02z1 i.enb_03z1 i.higheredu  i.soa_07z1 i.married i.CTPRVN_CODE) if agegroup==4 & goodqol==1,
vce(robust)
tebalance sum
est store highqol4

teffects ipw (depression0) (enb_05z1 i.sex  i.HOUSE_TY_CODE i.DONG_TY_CODE i.HOUSE_TY_CODE  i.basicsecurity
i.fma_25z1 i.smoked i.indirectsmoked i.sfa_02z3 i.drank i.pha_04z1 i.pha_07z1 i.phb_01z1 nua_01z2 oba_02z1
oba_03z1 i.diet goodteethhealth  i.gambled i.sca_01z1 healthcheckup stroke earlyperceivhrtatck i.hya_04z1
i.dia_04z1 i.sra_01z3 i.ira_01z1 i.ena_01a1 i.ena_01b1 i.ena_01c1 i.ena_01d1 i.ena_01e1 i.ena_01f1 i.ena_01g1
i.enb_01z1 i.enb_02z1 i.enb_03z1 i.higheredu  i.soa_07z1 i.married i.CTPRVN_CODE) if agegroup==4 & goodqol==0,
vce(robust)
tebalance sum
est store lowqol4


coefplot lowqol4 highqol4, yline(0) level(95) vertical ytitle(P(depression|X))




teffects ipw (depression0) (enb_05z1 i.sex  i.HOUSE_TY_CODE i.DONG_TY_CODE i.HOUSE_TY_CODE  i.basicsecurity
i.fma_25z1 i.smoked i.indirectsmoked i.sfa_02z3 i.drank i.pha_04z1 i.pha_07z1 i.phb_01z1 nua_01z2 oba_02z1
```

```
oba_03z1 i.diet goodteethhealth  i.gambled i.sca_01z1 healthcheckup stroke earlyperceivhrtatck i.hya_04z1
i.dia_04z1 i.sra_01z3 i.ira_01z1 i.ena_01a1 i.ena_01b1 i.ena_01c1 i.ena_01d1 i.ena_01e1 i.ena_01f1 i.ena_01g1
i.enb_01z1 i.enb_02z1 i.enb_03z1 i.higheredu  i.soa_07z1 i.married i.CTPRVN_CODE) if agegroup==5 & goodqol==1,
vce(robust)
tebalance sum
est store highqol5

teffects ipw (depression0) (enb_05z1 i.sex  i.HOUSE_TY_CODE i.DONG_TY_CODE i.HOUSE_TY_CODE  i.basicsecurity
i.fma_25z1 i.smoked i.indirectsmoked i.sfa_02z3 i.drank i.pha_04z1 i.pha_07z1 i.phb_01z1 nua_01z2 oba_02z1
oba_03z1 i.diet goodteethhealth  i.gambled i.sca_01z1 healthcheckup stroke earlyperceivhrtatck i.hya_04z1
i.dia_04z1 i.sra_01z3 i.ira_01z1 i.ena_01a1 i.ena_01b1 i.ena_01c1 i.ena_01d1 i.ena_01e1 i.ena_01f1 i.ena_01g1
i.enb_01z1 i.enb_02z1 i.enb_03z1 i.higheredu  i.soa_07z1 i.married i.CTPRVN_CODE) if agegroup==5 & goodqol==0,
vce(robust)
tebalance sum
est store lowqol5


coefplot lowqol5 highqol5, yline(0) level(95) vertical ytitle(P(depression|X))




teffects ipw (depression0) (enb_05z1 i.sex  i.HOUSE_TY_CODE i.DONG_TY_CODE i.HOUSE_TY_CODE  i.basicsecurity
i.fma_25z1 i.smoked i.indirectsmoked i.sfa_02z3 i.drank i.pha_04z1 i.pha_07z1 i.phb_01z1 nua_01z2 oba_02z1
oba_03z1 i.diet goodteethhealth  i.gambled i.sca_01z1 healthcheckup stroke earlyperceivhrtatck i.hya_04z1
i.dia_04z1 i.sra_01z3 i.ira_01z1 i.ena_01a1 i.ena_01b1 i.ena_01c1 i.ena_01d1 i.ena_01e1 i.ena_01f1 i.ena_01g1
i.enb_01z1 i.enb_02z1 i.enb_03z1 i.higheredu  i.soa_07z1 i.married i.CTPRVN_CODE) if agegroup==6 & goodqol==1,
vce(robust)
tebalance sum
est store highqol6

teffects ipw (depression0) (enb_05z1 i.sex  i.HOUSE_TY_CODE i.DONG_TY_CODE i.HOUSE_TY_CODE  i.basicsecurity
i.fma_25z1 i.smoked i.indirectsmoked i.sfa_02z3 i.drank i.pha_04z1 i.pha_07z1 i.phb_01z1 nua_01z2 oba_02z1
oba_03z1 i.diet goodteethhealth  i.gambled i.sca_01z1 healthcheckup stroke earlyperceivhrtatck i.hya_04z1
i.dia_04z1 i.sra_01z3 i.ira_01z1 i.ena_01a1 i.ena_01b1 i.ena_01c1 i.ena_01d1 i.ena_01e1 i.ena_01f1 i.ena_01g1
i.enb_01z1 i.enb_02z1 i.enb_03z1 i.higheredu  i.soa_07z1 i.married i.CTPRVN_CODE) if agegroup==6 & goodqol==0,
vce(robust)
tebalance sum
est store lowqol6


coefplot lowqol6 highqol6, yline(0) level(95) vertical ytitle(P(depression|X))




teffects ipw (depression0) (enb_05z1 i.sex  i.HOUSE_TY_CODE i.DONG_TY_CODE i.HOUSE_TY_CODE  i.basicsecurity
i.fma_25z1 i.smoked i.indirectsmoked i.sfa_02z3 i.drank i.pha_04z1 i.pha_07z1 i.phb_01z1 nua_01z2 oba_02z1
oba_03z1 i.diet goodteethhealth  i.gambled i.sca_01z1 healthcheckup stroke earlyperceivhrtatck i.hya_04z1
i.dia_04z1 i.sra_01z3 i.ira_01z1 i.ena_01a1 i.ena_01b1 i.ena_01c1 i.ena_01d1 i.ena_01e1 i.ena_01f1 i.ena_01g1
i.enb_01z1 i.enb_02z1 i.enb_03z1 i.higheredu  i.soa_07z1 i.married i.CTPRVN_CODE) if agegroup==7 & goodqol==1,
vce(robust)
tebalance sum
est store highqol7

teffects ipw (depression0) (enb_05z1 i.sex  i.HOUSE_TY_CODE i.DONG_TY_CODE i.HOUSE_TY_CODE  i.basicsecurity
i.fma_25z1 i.smoked i.indirectsmoked i.sfa_02z3 i.drank i.pha_04z1 i.pha_07z1 i.phb_01z1 nua_01z2 oba_02z1
oba_03z1 i.diet goodteethhealth  i.gambled i.sca_01z1 healthcheckup stroke earlyperceivhrtatck i.hya_04z1
i.dia_04z1 i.sra_01z3 i.ira_01z1 i.ena_01a1 i.ena_01b1 i.ena_01c1 i.ena_01d1 i.ena_01e1 i.ena_01f1 i.ena_01g1
i.enb_01z1 i.enb_02z1 i.enb_03z1 i.higheredu  i.soa_07z1 i.married i.CTPRVN_CODE) if agegroup==7 & goodqol==0,
vce(robust)
tebalance sum
est store lowqol7


coefplot lowqol7 highqol7, yline(0) level(95) vertical ytitle(P(depression|X))


coefplot lowqol2 highqol2 lowqol3 highqol3 lowqol4 highqol4 lowqol5 highqol5 lowqol6 highqol6 lowqol7 highqol7,
yline(0) level(95) vertical ytitle(P(depression|X))
*based on HRQoL


***********Stress


teffects ipw (stress) (enb_05z1 i.sex  i.HOUSE_TY_CODE i.DONG_TY_CODE i.HOUSE_TY_CODE  i.basicsecurity
i.fma_25z1 i.smoked i.indirectsmoked i.sfa_02z3 i.drank i.pha_04z1 i.pha_07z1 i.phb_01z1 nua_01z2 oba_02z1
oba_03z1 i.diet goodteethhealth  i.gambled i.sca_01z1 healthcheckup stroke earlyperceivhrtatck i.hya_04z1
i.dia_04z1 i.sra_01z3 i.ira_01z1 i.ena_01a1 i.ena_01b1 i.ena_01c1 i.ena_01d1 i.ena_01e1 i.ena_01f1 i.ena_01g1
```

```
i.enb_01z1 i.enb_02z1 i.enb_03z1 i.higheredu  i.soa_07z1 i.married i.CTPRVN_CODE) if agegroup<=2 & goodqol==1,
vce(robust)
tebalance sum
est store highqol2

teffects ipw (stress) (enb_05z1 i.sex  i.HOUSE_TY_CODE i.DONG_TY_CODE i.HOUSE_TY_CODE  i.basicsecurity
i.fma_25z1 i.smoked i.indirectsmoked i.sfa_02z3 i.drank i.pha_04z1 i.pha_07z1 i.phb_01z1 nua_01z2 oba_02z1
oba_03z1 i.diet goodteethhealth  i.gambled i.sca_01z1 healthcheckup stroke earlyperceivhrtatck i.hya_04z1
i.dia_04z1 i.sra_01z3 i.ira_01z1 i.ena_01a1 i.ena_01b1 i.ena_01c1 i.ena_01d1 i.ena_01e1 i.ena_01f1 i.ena_01g1
i.enb_01z1 i.enb_02z1 i.enb_03z1 i.higheredu  i.soa_07z1 i.married i.CTPRVN_CODE) if agegroup<=2 & goodqol==0,
vce(robust)
tebalance sum
est store lowqol2


coefplot lowqol2 highqol2, yline(0) level(95) vertical ytitle(P(stress|X))




teffects ipw (stress) (enb_05z1 i.sex  i.HOUSE_TY_CODE i.DONG_TY_CODE i.HOUSE_TY_CODE  i.basicsecurity
i.fma_25z1 i.smoked i.indirectsmoked i.sfa_02z3 i.drank i.pha_04z1 i.pha_07z1 i.phb_01z1 nua_01z2 oba_02z1
oba_03z1 i.diet goodteethhealth  i.gambled i.sca_01z1 healthcheckup stroke earlyperceivhrtatck i.hya_04z1
i.dia_04z1 i.sra_01z3 i.ira_01z1 i.ena_01a1 i.ena_01b1 i.ena_01c1 i.ena_01d1 i.ena_01e1 i.ena_01f1 i.ena_01g1
i.enb_01z1 i.enb_02z1 i.enb_03z1 i.higheredu  i.soa_07z1 i.married i.CTPRVN_CODE) if agegroup==3 & goodqol==1,
vce(robust)
tebalance sum
est store highqol3

teffects ipw (stress) (enb_05z1 i.sex  i.HOUSE_TY_CODE i.DONG_TY_CODE i.HOUSE_TY_CODE  i.basicsecurity
i.fma_25z1 i.smoked i.indirectsmoked i.sfa_02z3 i.drank i.pha_04z1 i.pha_07z1 i.phb_01z1 nua_01z2 oba_02z1
oba_03z1 i.diet goodteethhealth  i.gambled i.sca_01z1 healthcheckup stroke earlyperceivhrtatck i.hya_04z1
i.dia_04z1 i.sra_01z3 i.ira_01z1 i.ena_01a1 i.ena_01b1 i.ena_01c1 i.ena_01d1 i.ena_01e1 i.ena_01f1 i.ena_01g1
i.enb_01z1 i.enb_02z1 i.enb_03z1 i.higheredu  i.soa_07z1 i.married i.CTPRVN_CODE) if agegroup==3 & goodqol==0,
vce(robust)
tebalance sum
est store lowqol3


coefplot lowqol3 highqol3, yline(0) level(95) vertical ytitle(P(stress|X))



teffects ipw (stress) (enb_05z1 i.sex  i.HOUSE_TY_CODE i.DONG_TY_CODE i.HOUSE_TY_CODE  i.basicsecurity
i.fma_25z1 i.smoked i.indirectsmoked i.sfa_02z3 i.drank i.pha_04z1 i.pha_07z1 i.phb_01z1 nua_01z2 oba_02z1
oba_03z1 i.diet goodteethhealth  i.gambled i.sca_01z1 healthcheckup stroke earlyperceivhrtatck i.hya_04z1
i.dia_04z1 i.sra_01z3 i.ira_01z1 i.ena_01a1 i.ena_01b1 i.ena_01c1 i.ena_01d1 i.ena_01e1 i.ena_01f1 i.ena_01g1
i.enb_01z1 i.enb_02z1 i.enb_03z1 i.higheredu  i.soa_07z1 i.married i.CTPRVN_CODE) if agegroup==4 & goodqol==1,
vce(robust)
tebalance sum
est store highqol4

teffects ipw (stress) (enb_05z1 i.sex  i.HOUSE_TY_CODE i.DONG_TY_CODE i.HOUSE_TY_CODE  i.basicsecurity
i.fma_25z1 i.smoked i.indirectsmoked i.sfa_02z3 i.drank i.pha_04z1 i.pha_07z1 i.phb_01z1 nua_01z2 oba_02z1
oba_03z1 i.diet goodteethhealth  i.gambled i.sca_01z1 healthcheckup stroke earlyperceivhrtatck i.hya_04z1
i.dia_04z1 i.sra_01z3 i.ira_01z1 i.ena_01a1 i.ena_01b1 i.ena_01c1 i.ena_01d1 i.ena_01e1 i.ena_01f1 i.ena_01g1
i.enb_01z1 i.enb_02z1 i.enb_03z1 i.higheredu  i.soa_07z1 i.married i.CTPRVN_CODE) if agegroup==4 & goodqol==0,
vce(robust)
tebalance sum
est store lowqol4


coefplot lowqol4 highqol4, yline(0) level(95) vertical ytitle(P(stress|X))




teffects ipw (stress) (enb_05z1 i.sex  i.HOUSE_TY_CODE i.DONG_TY_CODE i.HOUSE_TY_CODE  i.basicsecurity
i.fma_25z1 i.smoked i.indirectsmoked i.sfa_02z3 i.drank i.pha_04z1 i.pha_07z1 i.phb_01z1 nua_01z2 oba_02z1
oba_03z1 i.diet goodteethhealth  i.gambled i.sca_01z1 healthcheckup stroke earlyperceivhrtatck i.hya_04z1
i.dia_04z1 i.sra_01z3 i.ira_01z1 i.ena_01a1 i.ena_01b1 i.ena_01c1 i.ena_01d1 i.ena_01e1 i.ena_01f1 i.ena_01g1
i.enb_01z1 i.enb_02z1 i.enb_03z1 i.higheredu  i.soa_07z1 i.married i.CTPRVN_CODE) if agegroup==5 & goodqol==1,
vce(robust)
tebalance sum
est store highqol5

teffects ipw (stress) (enb_05z1 i.sex  i.HOUSE_TY_CODE i.DONG_TY_CODE i.HOUSE_TY_CODE  i.basicsecurity
i.fma_25z1 i.smoked i.indirectsmoked i.sfa_02z3 i.drank i.pha_04z1 i.pha_07z1 i.phb_01z1 nua_01z2 oba_02z1
oba_03z1 i.diet goodteethhealth  i.gambled i.sca_01z1 healthcheckup stroke earlyperceivhrtatck i.hya_04z1
i.dia_04z1 i.sra_01z3 i.ira_01z1 i.ena_01a1 i.ena_01b1 i.ena_01c1 i.ena_01d1 i.ena_01e1 i.ena_01f1 i.ena_01g1
```

```
i.enb_01z1 i.enb_02z1 i.enb_03z1 i.higheredu  i.soa_07z1 i.married i.CTPRVN_CODE) if agegroup==5 & goodqol==0,
vce(robust)
tebalance sum
est store lowqol5


coefplot lowqol5 highqol5, yline(0) level(95) vertical ytitle(P(stress|X))




teffects ipw (stress) (enb_05z1 i.sex  i.HOUSE_TY_CODE i.DONG_TY_CODE i.HOUSE_TY_CODE  i.basicsecurity
i.fma_25z1 i.smoked i.indirectsmoked i.sfa_02z3 i.drank i.pha_04z1 i.pha_07z1 i.phb_01z1 nua_01z2 oba_02z1
oba_03z1 i.diet goodteethhealth  i.gambled i.sca_01z1 healthcheckup stroke earlyperceivhrtatck i.hya_04z1
i.dia_04z1 i.sra_01z3 i.ira_01z1 i.ena_01a1 i.ena_01b1 i.ena_01c1 i.ena_01d1 i.ena_01e1 i.ena_01f1 i.ena_01g1
i.enb_01z1 i.enb_02z1 i.enb_03z1 i.higheredu  i.soa_07z1 i.married i.CTPRVN_CODE) if agegroup==6 & goodqol==1,
vce(robust)
tebalance sum
est store highqol6

teffects ipw (stress) (enb_05z1 i.sex  i.HOUSE_TY_CODE i.DONG_TY_CODE i.HOUSE_TY_CODE  i.basicsecurity
i.fma_25z1 i.smoked i.indirectsmoked i.sfa_02z3 i.drank i.pha_04z1 i.pha_07z1 i.phb_01z1 nua_01z2 oba_02z1
oba_03z1 i.diet goodteethhealth  i.gambled i.sca_01z1 healthcheckup stroke earlyperceivhrtatck i.hya_04z1
i.dia_04z1 i.sra_01z3 i.ira_01z1 i.ena_01a1 i.ena_01b1 i.ena_01c1 i.ena_01d1 i.ena_01e1 i.ena_01f1 i.ena_01g1
i.enb_01z1 i.enb_02z1 i.enb_03z1 i.higheredu  i.soa_07z1 i.married i.CTPRVN_CODE) if agegroup==6 & goodqol==0,
vce(robust)
tebalance sum
est store lowqol6


coefplot lowqol6 highqol6, yline(0) level(95) vertical ytitle(P(stress|X))




teffects ipw (stress) (enb_05z1 i.sex  i.HOUSE_TY_CODE i.DONG_TY_CODE i.HOUSE_TY_CODE  i.basicsecurity
i.fma_25z1 i.smoked i.indirectsmoked i.sfa_02z3 i.drank i.pha_04z1 i.pha_07z1 i.phb_01z1 nua_01z2 oba_02z1
oba_03z1 i.diet goodteethhealth  i.gambled i.sca_01z1 healthcheckup stroke earlyperceivhrtatck i.hya_04z1
i.dia_04z1 i.sra_01z3 i.ira_01z1 i.ena_01a1 i.ena_01b1 i.ena_01c1 i.ena_01d1 i.ena_01e1 i.ena_01f1 i.ena_01g1
i.enb_01z1 i.enb_02z1 i.enb_03z1 i.higheredu  i.soa_07z1 i.married i.CTPRVN_CODE) if agegroup==7 & goodqol==1,
vce(robust)
tebalance sum
est store highqol7

teffects ipw (stress) (enb_05z1 i.sex  i.HOUSE_TY_CODE i.DONG_TY_CODE i.HOUSE_TY_CODE  i.basicsecurity
i.fma_25z1 i.smoked i.indirectsmoked i.sfa_02z3 i.drank i.pha_04z1 i.pha_07z1 i.phb_01z1 nua_01z2 oba_02z1
oba_03z1 i.diet goodteethhealth  i.gambled i.sca_01z1 healthcheckup stroke earlyperceivhrtatck i.hya_04z1
i.dia_04z1 i.sra_01z3 i.ira_01z1 i.ena_01a1 i.ena_01b1 i.ena_01c1 i.ena_01d1 i.ena_01e1 i.ena_01f1 i.ena_01g1
i.enb_01z1 i.enb_02z1 i.enb_03z1 i.higheredu  i.soa_07z1 i.married i.CTPRVN_CODE) if agegroup==7 & goodqol==0,
vce(robust)
tebalance sum
est store lowqol7


coefplot lowqol7 highqol7, yline(0) level(95) vertical ytitle(P(stress|X))

coefplot lowqol2 highqol2 lowqol3 highqol3 lowqol4 highqol4 lowqol5 highqol5 lowqol6 highqol6 lowqol7 highqol7,
yline(0) level(95) vertical ytitle(P(stress|X))





*********Main effects of Depression all combined



teffects ipw (depression0) (enb_05z1 goodqol i.sex  i.HOUSE_TY_CODE i.DONG_TY_CODE i.HOUSE_TY_CODE
i.basicsecurity  i.fma_25z1 i.smoked i.indirectsmoked i.sfa_02z3 i.drank i.pha_04z1 i.pha_07z1 i.phb_01z1
nua_01z2 oba_02z1 oba_03z1 i.diet goodteethhealth  i.gambled i.sca_01z1 healthcheckup stroke
earlyperceivhrtatck i.hya_04z1 i.dia_04z1 i.sra_01z3 i.ira_01z1 i.ena_01a1 i.ena_01b1 i.ena_01c1 i.ena_01d1
i.ena_01e1 i.ena_01f1 i.ena_01g1 i.enb_01z1 i.enb_02z1 i.enb_03z1 i.higheredu  i.soa_07z1 i.married
i.CTPRVN_CODE) if agegroup<=2  , vce(robust)
tebalance sum
est store network2

teffects ipw (depression0) (goodqol enb_05z1 i.sex  i.HOUSE_TY_CODE i.DONG_TY_CODE i.HOUSE_TY_CODE
i.basicsecurity  i.fma_25z1 i.smoked i.indirectsmoked i.sfa_02z3 i.drank i.pha_04z1 i.pha_07z1 i.phb_01z1
nua_01z2 oba_02z1 oba_03z1 i.diet goodteethhealth  i.gambled i.sca_01z1 healthcheckup stroke
```

```
earlyperceivhrtatck i.hya_04z1 i.dia_04z1 i.sra_01z3 i.ira_01z1 i.ena_01a1 i.ena_01b1 i.ena_01c1 i.ena_01d1
i.ena_01e1 i.ena_01f1 i.ena_01g1 i.enb_01z1 i.enb_02z1 i.enb_03z1 i.higheredu  i.soa_07z1 i.married
i.CTPRVN_CODE) if agegroup<=2  , vce(robust)
tebalance sum
est store goodqol2


teffects ipw (depression0) (enb_05z1 goodqol i.sex  i.HOUSE_TY_CODE i.DONG_TY_CODE i.HOUSE_TY_CODE
i.basicsecurity  i.fma_25z1 i.smoked i.indirectsmoked i.sfa_02z3 i.drank i.pha_04z1 i.pha_07z1 i.phb_01z1
nua_01z2 oba_02z1 oba_03z1 i.diet goodteethhealth  i.gambled i.sca_01z1 healthcheckup stroke
earlyperceivhrtatck i.hya_04z1 i.dia_04z1 i.sra_01z3 i.ira_01z1 i.ena_01a1 i.ena_01b1 i.ena_01c1 i.ena_01d1
i.ena_01e1 i.ena_01f1 i.ena_01g1 i.enb_01z1 i.enb_02z1 i.enb_03z1 i.higheredu  i.soa_07z1 i.married
i.CTPRVN_CODE) if agegroup==3  , vce(robust)
tebalance sum
est store network3

teffects ipw (depression0) (goodqol enb_05z1 i.sex  i.HOUSE_TY_CODE i.DONG_TY_CODE i.HOUSE_TY_CODE
i.basicsecurity  i.fma_25z1 i.smoked i.indirectsmoked i.sfa_02z3 i.drank i.pha_04z1 i.pha_07z1 i.phb_01z1
nua_01z2 oba_02z1 oba_03z1 i.diet goodteethhealth  i.gambled i.sca_01z1 healthcheckup stroke
earlyperceivhrtatck i.hya_04z1 i.dia_04z1 i.sra_01z3 i.ira_01z1 i.ena_01a1 i.ena_01b1 i.ena_01c1 i.ena_01d1
i.ena_01e1 i.ena_01f1 i.ena_01g1 i.enb_01z1 i.enb_02z1 i.enb_03z1 i.higheredu  i.soa_07z1 i.married
i.CTPRVN_CODE) if agegroup==3  , vce(robust)
tebalance sum
est store goodqol3


teffects ipw (depression0) (enb_05z1 goodqol i.sex  i.HOUSE_TY_CODE i.DONG_TY_CODE i.HOUSE_TY_CODE
i.basicsecurity  i.fma_25z1 i.smoked i.indirectsmoked i.sfa_02z3 i.drank i.pha_04z1 i.pha_07z1 i.phb_01z1
nua_01z2 oba_02z1 oba_03z1 i.diet goodteethhealth  i.gambled i.sca_01z1 healthcheckup stroke
earlyperceivhrtatck i.hya_04z1 i.dia_04z1 i.sra_01z3 i.ira_01z1 i.ena_01a1 i.ena_01b1 i.ena_01c1 i.ena_01d1
i.ena_01e1 i.ena_01f1 i.ena_01g1 i.enb_01z1 i.enb_02z1 i.enb_03z1 i.higheredu  i.soa_07z1 i.married
i.CTPRVN_CODE) if agegroup==4  , vce(robust)
tebalance sum
est store network4

teffects ipw (depression0) (goodqol enb_05z1 i.sex  i.HOUSE_TY_CODE i.DONG_TY_CODE i.HOUSE_TY_CODE
i.basicsecurity  i.fma_25z1 i.smoked i.indirectsmoked i.sfa_02z3 i.drank i.pha_04z1 i.pha_07z1 i.phb_01z1
nua_01z2 oba_02z1 oba_03z1 i.diet goodteethhealth  i.gambled i.sca_01z1 healthcheckup stroke
earlyperceivhrtatck i.hya_04z1 i.dia_04z1 i.sra_01z3 i.ira_01z1 i.ena_01a1 i.ena_01b1 i.ena_01c1 i.ena_01d1
i.ena_01e1 i.ena_01f1 i.ena_01g1 i.enb_01z1 i.enb_02z1 i.enb_03z1 i.higheredu  i.soa_07z1 i.married
i.CTPRVN_CODE) if agegroup==4  , vce(robust)
tebalance sum
est store goodqol4


teffects ipw (depression0) (enb_05z1 goodqol i.sex  i.HOUSE_TY_CODE i.DONG_TY_CODE i.HOUSE_TY_CODE
i.basicsecurity  i.fma_25z1 i.smoked i.indirectsmoked i.sfa_02z3 i.drank i.pha_04z1 i.pha_07z1 i.phb_01z1
nua_01z2 oba_02z1 oba_03z1 i.diet goodteethhealth  i.gambled i.sca_01z1 healthcheckup stroke
earlyperceivhrtatck i.hya_04z1 i.dia_04z1 i.sra_01z3 i.ira_01z1 i.ena_01a1 i.ena_01b1 i.ena_01c1 i.ena_01d1
i.ena_01e1 i.ena_01f1 i.ena_01g1 i.enb_01z1 i.enb_02z1 i.enb_03z1 i.higheredu  i.soa_07z1 i.married
i.CTPRVN_CODE) if agegroup==5  , vce(robust)
tebalance sum
est store network5

teffects ipw (depression0) (goodqol enb_05z1 i.sex  i.HOUSE_TY_CODE i.DONG_TY_CODE i.HOUSE_TY_CODE
i.basicsecurity  i.fma_25z1 i.smoked i.indirectsmoked i.sfa_02z3 i.drank i.pha_04z1 i.pha_07z1 i.phb_01z1
nua_01z2 oba_02z1 oba_03z1 i.diet goodteethhealth  i.gambled i.sca_01z1 healthcheckup stroke
earlyperceivhrtatck i.hya_04z1 i.dia_04z1 i.sra_01z3 i.ira_01z1 i.ena_01a1 i.ena_01b1 i.ena_01c1 i.ena_01d1
i.ena_01e1 i.ena_01f1 i.ena_01g1 i.enb_01z1 i.enb_02z1 i.enb_03z1 i.higheredu  i.soa_07z1 i.married
i.CTPRVN_CODE) if agegroup==5 , vce(robust)
tebalance sum
est store goodqol5


teffects ipw (depression0) (enb_05z1 goodqol i.sex  i.HOUSE_TY_CODE i.DONG_TY_CODE i.HOUSE_TY_CODE
i.basicsecurity  i.fma_25z1 i.smoked i.indirectsmoked i.sfa_02z3 i.drank i.pha_04z1 i.pha_07z1 i.phb_01z1
nua_01z2 oba_02z1 oba_03z1 i.diet goodteethhealth  i.gambled i.sca_01z1 healthcheckup stroke
earlyperceivhrtatck i.hya_04z1 i.dia_04z1 i.sra_01z3 i.ira_01z1 i.ena_01a1 i.ena_01b1 i.ena_01c1 i.ena_01d1
i.ena_01e1 i.ena_01f1 i.ena_01g1 i.enb_01z1 i.enb_02z1 i.enb_03z1 i.higheredu  i.soa_07z1 i.married
i.CTPRVN_CODE) if agegroup==6  , vce(robust)
tebalance sum
est store network6

teffects ipw (depression0) (goodqol enb_05z1 i.sex  i.HOUSE_TY_CODE i.DONG_TY_CODE i.HOUSE_TY_CODE
i.basicsecurity  i.fma_25z1 i.smoked i.indirectsmoked i.sfa_02z3 i.drank i.pha_04z1 i.pha_07z1 i.phb_01z1
nua_01z2 oba_02z1 oba_03z1 i.diet goodteethhealth  i.gambled i.sca_01z1 healthcheckup stroke
earlyperceivhrtatck i.hya_04z1 i.dia_04z1 i.sra_01z3 i.ira_01z1 i.ena_01a1 i.ena_01b1 i.ena_01c1 i.ena_01d1
i.ena_01e1 i.ena_01f1 i.ena_01g1 i.enb_01z1 i.enb_02z1 i.enb_03z1 i.higheredu  i.soa_07z1 i.married
i.CTPRVN_CODE) if agegroup==6 , vce(robust)
tebalance sum
est store goodqol6
```

```
teffects ipw (depression0) (enb_05z1 goodqol i.sex  i.HOUSE_TY_CODE i.DONG_TY_CODE i.HOUSE_TY_CODE
i.basicsecurity  i.fma_25z1 i.smoked i.indirectsmoked i.sfa_02z3 i.drank i.pha_04z1 i.pha_07z1 i.phb_01z1
nua_01z2 oba_02z1 oba_03z1 i.diet goodteethhealth  i.gambled i.sca_01z1 healthcheckup stroke
earlyperceivhrtatck i.hya_04z1 i.dia_04z1 i.sra_01z3 i.ira_01z1 i.ena_01a1 i.ena_01b1 i.ena_01c1 i.ena_01d1
i.ena_01e1 i.ena_01f1 i.ena_01g1 i.enb_01z1 i.enb_02z1 i.enb_03z1 i.higheredu  i.soa_07z1 i.married
i.CTPRVN_CODE) if agegroup==7 , vce(robust)
tebalance sum
est store network7

teffects ipw (depression0) (goodqol enb_05z1 i.sex  i.HOUSE_TY_CODE i.DONG_TY_CODE i.HOUSE_TY_CODE
i.basicsecurity  i.fma_25z1 i.smoked i.indirectsmoked i.sfa_02z3 i.drank i.pha_04z1 i.pha_07z1 i.phb_01z1
nua_01z2 oba_02z1 oba_03z1 i.diet goodteethhealth  i.gambled i.sca_01z1 healthcheckup stroke
earlyperceivhrtatck i.hya_04z1 i.dia_04z1 i.sra_01z3 i.ira_01z1 i.ena_01a1 i.ena_01b1 i.ena_01c1 i.ena_01d1
i.ena_01e1 i.ena_01f1 i.ena_01g1 i.enb_01z1 i.enb_02z1 i.enb_03z1 i.higheredu  i.soa_07z1 i.married
i.CTPRVN_CODE) if agegroup==7, vce(robust)
tebalance sum
est store goodqol7



coefplot network2 goodqol2 network3 goodqol3 network4 goodqol4 network5 goodqol5 network6 goodqol6 network7
goodqol7, yline(0) level(95) vertical ytitle(P(depression|X))


********Main effects of stress all combined
teffects ipw (stress) (enb_05z1 goodqol i.sex  i.HOUSE_TY_CODE i.DONG_TY_CODE i.HOUSE_TY_CODE  i.basicsecurity
i.fma_25z1 i.smoked i.indirectsmoked i.sfa_02z3 i.drank i.pha_04z1 i.pha_07z1 i.phb_01z1 nua_01z2 oba_02z1
oba_03z1 i.diet goodteethhealth  i.gambled i.sca_01z1 healthcheckup stroke earlyperceivhrtatck i.hya_04z1
i.dia_04z1 i.sra_01z3 i.ira_01z1 i.ena_01a1 i.ena_01b1 i.ena_01c1 i.ena_01d1 i.ena_01e1 i.ena_01f1 i.ena_01g1
i.enb_01z1 i.enb_02z1 i.enb_03z1 i.higheredu  i.soa_07z1 i.married i.CTPRVN_CODE) if agegroup<=2  , vce(robust)
tebalance sum
est store network2

teffects ipw (stress) (goodqol enb_05z1 i.sex  i.HOUSE_TY_CODE i.DONG_TY_CODE i.HOUSE_TY_CODE  i.basicsecurity
i.fma_25z1 i.smoked i.indirectsmoked i.sfa_02z3 i.drank i.pha_04z1 i.pha_07z1 i.phb_01z1 nua_01z2 oba_02z1
oba_03z1 i.diet goodteethhealth  i.gambled i.sca_01z1 healthcheckup stroke earlyperceivhrtatck i.hya_04z1
i.dia_04z1 i.sra_01z3 i.ira_01z1 i.ena_01a1 i.ena_01b1 i.ena_01c1 i.ena_01d1 i.ena_01e1 i.ena_01f1 i.ena_01g1
i.enb_01z1 i.enb_02z1 i.enb_03z1 i.higheredu  i.soa_07z1 i.married i.CTPRVN_CODE) if agegroup<=2  , vce(robust)
tebalance sum
est store goodqol2


teffects ipw (stress) (enb_05z1 goodqol i.sex  i.HOUSE_TY_CODE i.DONG_TY_CODE i.HOUSE_TY_CODE  i.basicsecurity
i.fma_25z1 i.smoked i.indirectsmoked i.sfa_02z3 i.drank i.pha_04z1 i.pha_07z1 i.phb_01z1 nua_01z2 oba_02z1
oba_03z1 i.diet goodteethhealth  i.gambled i.sca_01z1 healthcheckup stroke earlyperceivhrtatck i.hya_04z1
i.dia_04z1 i.sra_01z3 i.ira_01z1 i.ena_01a1 i.ena_01b1 i.ena_01c1 i.ena_01d1 i.ena_01e1 i.ena_01f1 i.ena_01g1
i.enb_01z1 i.enb_02z1 i.enb_03z1 i.higheredu  i.soa_07z1 i.married i.CTPRVN_CODE) if agegroup==3  , vce(robust)
tebalance sum
est store network3

teffects ipw (stress) (goodqol enb_05z1 i.sex  i.HOUSE_TY_CODE i.DONG_TY_CODE i.HOUSE_TY_CODE  i.basicsecurity
i.fma_25z1 i.smoked i.indirectsmoked i.sfa_02z3 i.drank i.pha_04z1 i.pha_07z1 i.phb_01z1 nua_01z2 oba_02z1
oba_03z1 i.diet goodteethhealth  i.gambled i.sca_01z1 healthcheckup stroke earlyperceivhrtatck i.hya_04z1
i.dia_04z1 i.sra_01z3 i.ira_01z1 i.ena_01a1 i.ena_01b1 i.ena_01c1 i.ena_01d1 i.ena_01e1 i.ena_01f1 i.ena_01g1
i.enb_01z1 i.enb_02z1 i.enb_03z1 i.higheredu  i.soa_07z1 i.married i.CTPRVN_CODE) if agegroup==3  , vce(robust)
tebalance sum
est store goodqol3


teffects ipw (stress) (enb_05z1 goodqol i.sex  i.HOUSE_TY_CODE i.DONG_TY_CODE i.HOUSE_TY_CODE  i.basicsecurity
i.fma_25z1 i.smoked i.indirectsmoked i.sfa_02z3 i.drank i.pha_04z1 i.pha_07z1 i.phb_01z1 nua_01z2 oba_02z1
oba_03z1 i.diet goodteethhealth  i.gambled i.sca_01z1 healthcheckup stroke earlyperceivhrtatck i.hya_04z1
i.dia_04z1 i.sra_01z3 i.ira_01z1 i.ena_01a1 i.ena_01b1 i.ena_01c1 i.ena_01d1 i.ena_01e1 i.ena_01f1 i.ena_01g1
i.enb_01z1 i.enb_02z1 i.enb_03z1 i.higheredu  i.soa_07z1 i.married i.CTPRVN_CODE) if agegroup==4  , vce(robust)
tebalance sum
est store network4

teffects ipw (stress) (goodqol enb_05z1 i.sex  i.HOUSE_TY_CODE i.DONG_TY_CODE i.HOUSE_TY_CODE  i.basicsecurity
i.fma_25z1 i.smoked i.indirectsmoked i.sfa_02z3 i.drank i.pha_04z1 i.pha_07z1 i.phb_01z1 nua_01z2 oba_02z1
oba_03z1 i.diet goodteethhealth  i.gambled i.sca_01z1 healthcheckup stroke earlyperceivhrtatck i.hya_04z1
i.dia_04z1 i.sra_01z3 i.ira_01z1 i.ena_01a1 i.ena_01b1 i.ena_01c1 i.ena_01d1 i.ena_01e1 i.ena_01f1 i.ena_01g1
i.enb_01z1 i.enb_02z1 i.enb_03z1 i.higheredu  i.soa_07z1 i.married i.CTPRVN_CODE) if agegroup==4  , vce(robust)
tebalance sum
est store goodqol4


teffects ipw (stress) (enb_05z1 goodqol i.sex  i.HOUSE_TY_CODE i.DONG_TY_CODE i.HOUSE_TY_CODE  i.basicsecurity
i.fma_25z1 i.smoked i.indirectsmoked i.sfa_02z3 i.drank i.pha_04z1 i.pha_07z1 i.phb_01z1 nua_01z2 oba_02z1
oba_03z1 i.diet goodteethhealth  i.gambled i.sca_01z1 healthcheckup stroke earlyperceivhrtatck i.hya_04z1
```

```
i.dia_04z1 i.sra_01z3 i.ira_01z1 i.ena_01a1 i.ena_01b1 i.ena_01c1 i.ena_01d1 i.ena_01e1 i.ena_01f1 i.ena_01g1
i.enb_01z1 i.enb_02z1 i.enb_03z1 i.higheredu  i.soa_07z1 i.married i.CTPRVN_CODE) if agegroup==5  , vce(robust)
tebalance sum
est store network5

teffects ipw (stress) (goodqol enb_05z1 i.sex  i.HOUSE_TY_CODE i.DONG_TY_CODE i.HOUSE_TY_CODE  i.basicsecurity
i.fma_25z1 i.smoked i.indirectsmoked i.sfa_02z3 i.drank i.pha_04z1 i.pha_07z1 i.phb_01z1 nua_01z2 oba_02z1
oba_03z1 i.diet goodteethhealth  i.gambled i.sca_01z1 healthcheckup stroke earlyperceivhrtatck i.hya_04z1
i.dia_04z1 i.sra_01z3 i.ira_01z1 i.ena_01a1 i.ena_01b1 i.ena_01c1 i.ena_01d1 i.ena_01e1 i.ena_01f1 i.ena_01g1
i.enb_01z1 i.enb_02z1 i.enb_03z1 i.higheredu  i.soa_07z1 i.married i.CTPRVN_CODE) if agegroup==5 , vce(robust)
tebalance sum
est store goodqol5


teffects ipw (stress) (enb_05z1 goodqol i.sex  i.HOUSE_TY_CODE i.DONG_TY_CODE i.HOUSE_TY_CODE  i.basicsecurity
i.fma_25z1 i.smoked i.indirectsmoked i.sfa_02z3 i.drank i.pha_04z1 i.pha_07z1 i.phb_01z1 nua_01z2 oba_02z1
oba_03z1 i.diet goodteethhealth  i.gambled i.sca_01z1 healthcheckup stroke earlyperceivhrtatck i.hya_04z1
i.dia_04z1 i.sra_01z3 i.ira_01z1 i.ena_01a1 i.ena_01b1 i.ena_01c1 i.ena_01d1 i.ena_01e1 i.ena_01f1 i.ena_01g1
i.enb_01z1 i.enb_02z1 i.enb_03z1 i.higheredu  i.soa_07z1 i.married i.CTPRVN_CODE) if agegroup==6  , vce(robust)
tebalance sum
est store network6

teffects ipw (stress) (goodqol enb_05z1 i.sex  i.HOUSE_TY_CODE i.DONG_TY_CODE i.HOUSE_TY_CODE  i.basicsecurity
i.fma_25z1 i.smoked i.indirectsmoked i.sfa_02z3 i.drank i.pha_04z1 i.pha_07z1 i.phb_01z1 nua_01z2 oba_02z1
oba_03z1 i.diet goodteethhealth  i.gambled i.sca_01z1 healthcheckup stroke earlyperceivhrtatck i.hya_04z1
i.dia_04z1 i.sra_01z3 i.ira_01z1 i.ena_01a1 i.ena_01b1 i.ena_01c1 i.ena_01d1 i.ena_01e1 i.ena_01f1 i.ena_01g1
i.enb_01z1 i.enb_02z1 i.enb_03z1 i.higheredu  i.soa_07z1 i.married i.CTPRVN_CODE) if agegroup==6 , vce(robust)
tebalance sum
est store goodqol6

teffects ipw (stress) (enb_05z1 goodqol i.sex  i.HOUSE_TY_CODE i.DONG_TY_CODE i.HOUSE_TY_CODE  i.basicsecurity
i.fma_25z1 i.smoked i.indirectsmoked i.sfa_02z3 i.drank i.pha_04z1 i.pha_07z1 i.phb_01z1 nua_01z2 oba_02z1
oba_03z1 i.diet goodteethhealth  i.gambled i.sca_01z1 healthcheckup stroke earlyperceivhrtatck i.hya_04z1
i.dia_04z1 i.sra_01z3 i.ira_01z1 i.ena_01a1 i.ena_01b1 i.ena_01c1 i.ena_01d1 i.ena_01e1 i.ena_01f1 i.ena_01g1
i.enb_01z1 i.enb_02z1 i.enb_03z1 i.higheredu  i.soa_07z1 i.married i.CTPRVN_CODE) if agegroup==7 , vce(robust)
tebalance sum
est store network7

teffects ipw (stress) (goodqol enb_05z1 i.sex  i.HOUSE_TY_CODE i.DONG_TY_CODE i.HOUSE_TY_CODE  i.basicsecurity
i.fma_25z1 i.smoked i.indirectsmoked i.sfa_02z3 i.drank i.pha_04z1 i.pha_07z1 i.phb_01z1 nua_01z2 oba_02z1
oba_03z1 i.diet goodteethhealth  i.gambled i.sca_01z1 healthcheckup stroke earlyperceivhrtatck i.hya_04z1
i.dia_04z1 i.sra_01z3 i.ira_01z1 i.ena_01a1 i.ena_01b1 i.ena_01c1 i.ena_01d1 i.ena_01e1 i.ena_01f1 i.ena_01g1
i.enb_01z1 i.enb_02z1 i.enb_03z1 i.higheredu  i.soa_07z1 i.married i.CTPRVN_CODE) if agegroup==7, vce(robust)
tebalance sum
est store goodqol7



coefplot network2 goodqol2 network3 goodqol3 network4 goodqol4 network5 goodqol5 network6 goodqol6 network7
goodqol7, yline(0) level(95) vertical ytitle(P(stress|X))


*******Figures: Online Supplementary Appendix********
replace agegroup=2 if agegroup==1
*Now, agegroup=2 means those who are in their 20s or below. For regerssion models and IPW models shown above,
they were coded differently from 1 -7. Now, we use 2-7, where 2 means those who are in their 20s or below


lab define   goodqolcombo 0 "HRQoL=No" 1 "HRQoL=High"
lab value goodqol goodqolcombo
lab define agegroup2  2 "-20s" 3 "30s" 4 "40s" 5 "50s" 6 "60s" 7 "70s+"
lab value agegroup agegroup2
codebook agegroup


foreach ag of numlist 2/7 {
    foreach q of numlist 0 1 {
        quietly: areg depression0 i.goodqol##i.enb_05z1##i.agegroup i.sex  i.HOUSE_TY_CODE i.DONG_TY_CODE
i.HOUSE_TY_CODE  i.basicsecurity  i.fma_25z1 i.smoked i.indirectsmoked i.sfa_02z3 i.drank i.pha_04z1 i.pha_07z1
i.phb_01z1 nua_01z2 oba_02z1 oba_03z1 i.diet goodteethhealth  i.gambled i.sca_01z1 healthcheckup stroke
earlyperceivhrtatck i.hya_04z1 i.dia_04z1 i.sra_01z3 i.ira_01z1 i.ena_01a1 i.ena_01b1 i.ena_01c1 i.ena_01d1
i.ena_01e1 i.ena_01f1 i.ena_01g1 i.enb_01z1 i.enb_02z1 i.enb_03z1 i.higheredu  i.soa_07z1 i.married  ,
absorb(signgu_code) vce(cluster signgu_code)
      quietly:  margins enb_05z1, at(goodqol=(0 1) agegroup=(2(1)7)) saving(m`ag'_q`q'_1, replace)
    }
}


combomarginsplot m2_q0_1 m2_q1_1 m3_q0_1 m3_q1_1 m4_q0_1 m4_q1_1 m5_q0_1 m5_q1_1 m6_q0_1 m6_q1_1 m7_q0_1
m7_q1_1 ,   bydimension(  agegroup)   xlabel(0 "No" 1 "Yes") ytitle("P(depression|X)") xtitle("Social
networking (at least once per month)")   legend(off)
```

```
foreach ag of numlist 2/7 {
    foreach q of numlist 0 1 {
        quietly: areg stress i.goodqol##i.enb_05z1##i.agegroup i.sex  i.HOUSE_TY_CODE i.DONG_TY_CODE
i.HOUSE_TY_CODE  i.basicsecurity  i.fma_25z1 i.smoked i.indirectsmoked i.sfa_02z3 i.drank i.pha_04z1 i.pha_07z1
i.phb_01z1 nua_01z2 oba_02z1 oba_03z1 i.diet goodteethhealth  i.gambled i.sca_01z1 healthcheckup stroke
earlyperceivhrtatck i.hya_04z1 i.dia_04z1 i.sra_01z3 i.ira_01z1 i.ena_01a1 i.ena_01b1 i.ena_01c1 i.ena_01d1
i.ena_01e1 i.ena_01f1 i.ena_01g1 i.enb_01z1 i.enb_02z1 i.enb_03z1 i.higheredu  i.soa_07z1 i.married  ,
absorb(signgu_code) vce(cluster signgu_code)
       quietly: margins enb_05z1, at(goodqol=(0 1) agegroup=(2(1)7)) saving(m`ag'_q`q'_2, replace)
    }
}


combomarginsplot m2_q0_2 m2_q1_2 m3_q0_2 m3_q1_2 m4_q0_2 m4_q1_2 m5_q0_2 m5_q1_2 m6_q0_2 m6_q1_2 m7_q0_2
m7_q1_2 ,   bydimension(  agegroup)   xlabel(0 "No" 1 "Yes") ytitle("P(stress|X)") xtitle("Social networking
(at least once per month)")   legend(off)





********Figures 1 and 2********

/*
replace agegroup=2 if agegroup==1
*Now, agegroup=2 means those who are in their 20s or below. For regerssion models, they were coded differently
from 1 -7. Now, we use 2-7, where 2 means those who are in their 20s or below

lab define   goodqolcombo 0 "HRQoL=No" 1 "HRQoL=High"
lab value goodqol goodqolcombo
lab define agegroup2  2 "-20s" 3 "30s" 4 "40s" 5 "50s" 6 "60s" 7 "70s+"
lab value agegroup agegroup2
*/

*****Figure 1******

codebook agegroup



quietly: areg depression0 i.goodqol##i.enb_05z1##i.agegroup i.sex  i.HOUSE_TY_CODE i.DONG_TY_CODE
i.HOUSE_TY_CODE  i.basicsecurity  i.fma_25z1 i.smoked i.indirectsmoked i.sfa_02z3 i.drank i.pha_04z1 i.pha_07z1
i.phb_01z1 nua_01z2 oba_02z1 oba_03z1 i.diet goodteethhealth  i.gambled i.sca_01z1 healthcheckup stroke
earlyperceivhrtatck i.hya_04z1 i.dia_04z1 i.sra_01z3 i.ira_01z1 i.ena_01a1 i.ena_01b1 i.ena_01c1 i.ena_01d1
i.ena_01e1 i.ena_01f1 i.ena_01g1 i.enb_01z1 i.enb_02z1 i.enb_03z1 i.higheredu  i.soa_07z1 i.married  ,
absorb(signgu_code) vce(cluster signgu_code)

* Step 1: Average marginal effect of networking at QoL=0 and QoL=1 per age
margins, dydx(enb_05z1) at(goodqol=(0 1)) over(agegroup)


* Plot
marginsplot, xdimension(agegroup) recast(connected) recastci(rcap)   ytitle("AME of social networking on
depression")    xtitle("Age group")   title("") level(95)




quietly: areg stress i.goodqol##i.enb_05z1##i.agegroup i.sex  i.HOUSE_TY_CODE i.DONG_TY_CODE i.HOUSE_TY_CODE
i.basicsecurity  i.fma_25z1 i.smoked i.indirectsmoked i.sfa_02z3 i.drank i.pha_04z1 i.pha_07z1 i.phb_01z1
nua_01z2 oba_02z1 oba_03z1 i.diet goodteethhealth  i.gambled i.sca_01z1 healthcheckup stroke
earlyperceivhrtatck i.hya_04z1 i.dia_04z1 i.sra_01z3 i.ira_01z1 i.ena_01a1 i.ena_01b1 i.ena_01c1 i.ena_01d1
i.ena_01e1 i.ena_01f1 i.ena_01g1 i.enb_01z1 i.enb_02z1 i.enb_03z1 i.higheredu  i.soa_07z1 i.married  ,
absorb(signgu_code) vce(cluster signgu_code)

* Step 1: Average marginal effect of networking at QoL=0 and QoL=1 per age
margins, dydx(enb_05z1) at(goodqol=(0 1)) over(agegroup)



* Plot
marginsplot, xdimension(agegroup) recast(connected) recastci(rcap)   ytitle("AME of social networking on
stress")    xtitle("Age group")   title("") level(95)
```

```
clear allss
```
